# Phenolic Assesment of *Uncaria tomentosa* L. (Cat’s Claw): Leaves, Stem, Bark and Wood Extracts

**DOI:** 10.3390/molecules201219875

**Published:** 2015-12-18

**Authors:** Mirtha Navarro Hoyos, Fernando Sánchez-Patán, Renato Murillo Masis, Pedro J. Martín-Álvarez, William Zamora Ramirez, Maria J. Monagas, Begoña Bartolomé

**Affiliations:** 1 Department of Chemistry at the University of Costa Rica (UCR), Sede Rodrigo Facio, San Pedro de Montes de Oca, San José 2060, Costa Rica; mirtha.navarro@ucr.ac.cr (M.N.H.); renato.murillo@ucr.ac.cr (R.M.M.); william.zamora@ucr.ac.cr (W.Z.R.); 2 Department of Food Biotechnology and Microbiology, Institute of Food Science Research (CIAL), CSIC-UAM, C/Nicolás Cabrera 9, Madrid 28049, Spain; f.s.patan@csic.es (F.S.-P.); pedroj.martin.alvarez@csic.es (P.J.M.-Á.); MJM@USP.org (M.J.M.)

**Keywords:** *Uncaria tomentosa* L., alkaloids, UPLC, TQ-ESI/MS, flavonoids, ^13^C-NMR, proanthocyanidins, propelargonidins, alkaloids

## Abstract

The phenolic composition of extracts from *Uncaria tomentosa* L. from different regions of Costa Rica was studied using advanced analytical techniques such as UPLC/TQ-ESI-MS and ^13^C-NMR. Samples from leaves, stems, bark and wood (*n* = 22) were subjected to extraction to obtain phenolic and alkaloid extracts, separately. Comparatively, higher values of total phenolic content were observed for leaves, stems and bark (225–494 gallic acid equivalents/g) than for wood extracts (40–167 gallic acid equivalents/g). A total of 32 non-flavonoid and flavonoid compounds were identified in the phenolic extracts: hydroxybenzoic acids (benzoic, salicylic, 4-hydroxybenzoic, prochatechuic, gallic, syringic and vanillic acids), hydroxycinnamic acids (p-coumaric, caffeic, ferulic and isoferulic acids), flavan-3-ols monomers [(+)-catechin and (−)-epicatechin)], procyanidin dimers (B1, B2, B3, B4, B5, B7 and two other of unknown structure) and trimers (C1, T2 and one of unknown structure), flavalignans (four unknown structures pertaining to the cinchonain family) and propelargonidin dimers (four unknown structures, reported for the first time in *U. tomentosa*). Additionally, alkaloid extracts obtained from the plant residue after phenolic extraction exhibited a content of tetracyclic and pentacyclic alkaloids ranging between 95 and 275 mg/100 g of dry material for bark extracts, and between 30 and 704 mg/100 g for leaves extracts. In addition, a minor alkaloid was isolated and characterized, namely 18,19-dehydrocorynoxinoic acid. Our results confirmed the feasibility of *U. tomentosa* as a suitable raw material for obtaining phenolic- and alkaloid-rich extracts of potential interest.

## 1. Introduction

*Uncaria tomentosa* L., also known as cat’s claw, is a creeper plant typical of the rainy tropical forest that belongs to the Rubiaceae family. It is naturally distributed from Peru to Belize, and traditionally used as a medicinal plant. In the last two decades, around 50 compounds have been isolated from *U. tomentosa*, from which around 35 can be considered exclusive to this species. These compounds include alkaloids, terpenes, quinolic acid glycosides and phenolic compounds, among them coumarins [1]. The scientific interest for this plant has increased after the confirmation of its immunomodulatory properties mainly attributed to its alkaloid content [1]. Moreover, there is a certain body of scientific evidence that demonstrates a wide spectra of bioactivities for *U. tomentosa* extracts, including immunomodulatory, antioxidant, antiviral, anti-inflammatory [2], also suggesting that these effects are due to synergism with other chemical compounds present in the plant [3]. In fact, some studies indicated that anti-inflammatory activity of *U. tomentosa* extracts is independent from their alkaloid contents [4,5].

Other studies suggest that phenolic compounds could be responsible for some pharmacologic effects of *U. tomentosa*, for instance, its antioxidant properties [6,7]. However, in general, phenolic composition of this plant has been scarcely studied and there are no reports on phenolic determinations using modern analytical techniques such as mass spectrometry. The presence of non-flavonoids, mainly hydroxycinnamic acids such as caffeic and chlorogenic acids, in *U. tomentosa* has been described [8,9,10]. Flavonoids present in this plant mainly include flavonols (*i.e.*, quercetin and miricetin derivatives) and flavan-3-ols [6,8,9]. In relation to this latter group, the monomers (+)-catechin and (−)-epicatechin, and the dimers procyanidins B1, B2 and B4 have been described in different preparations from *U. tomentosa* [9,11,12]. In addition, and of great interest is its content of flavalignans, *i.e.*, epichatechins substituted with phenylpropanoids, such as cinchonains 1a and 1b [13], which are not that common in nature but are present in medicinal plants such as *Eriobotrya japonica* [14]. In this paper, we have obtained extracts from different parts (leaves, stems, bark and wood) of *U. tomentosa* plants collected in different zones of Costa Rica, and we have characterize them using UPLC-DAD-ESI-TQ MS techniques complemented with ^13^C-NMR. Additionally, alkaloids extracts were prepared from the residues of *U. tomentosa* plants after phenolic extraction, and they were characterized in detail.

## 2. Results and Discussion

### 2.1. Phenolic Yield and Total Phenolic Content in U. tomentosa Extracts

The purification of the polar fraction with solvents of low and medium polarity allowed us to obtain phenolic enriched fractions with yields as shown in Table 1. Leaves gave the higher yields for all plants with an average of 11.7%, followed by bark with an average of 5.4% while wood and stems had a similar lower yield average of 3.2% and 2.9%, respectively. The total phenolic contents of the different extracts resulted in lower values for wood extracts (40–167 gallic acid equivalents/g) and higher values for leaves, stems and bark (225–494 gallic acid equivalents/g) being values varying depending on plant origin, for instance bark samples in the case of the location of Sarapiqui (S1), leaves samples in Palacios (P1) and stem samples in Asomat (AS).

**Table 1:** Extraction yield and total phenolic content of extracts from *U. tomentosa*.

Location	Sample	Extraction Yield (%) ^1^	Total Phenolic Content (mg/g) ^2^
Asomat (AS)	Leaves (AS-H)	20.6	356.3 ± 38.3
	Stems (AS-T)	3	460.0 ± 36.7
	Bark (AS-C)	3.2	315.6 ± 3.1
	Wood (AS-M)	2.3	95.7 ± 4.3
Los Chiles 1 (L1)	Leaves (L1-H)	10.4	431.7 ± 11.5
	Stems (L1-T)	4.4	247.7 ± 20.5
Los Chiles 2 (L2)	Leaves (L2-H)	5.7	426.3 ± 5.3
Palacios (P1)	Leaves (P1-H)	14.9	371.8 ± 0.7
	Stems (P1-T)	3	283.3 ± 3.3
	Bark (P1-C)	5.2	282.7 ± 13.5
	Wood (P1-M)	4.6	80.4 ± 4.3
Fortuna 1 (F1)	Leaves (F1-H)	7.7	336.4 ± 23.4
	Stems (F1-T)	2.2	236.4 ± 22.7
	Wood (F1-M)	3.7	40.5 ± 2.7
Fortuna 2 (F2)	Leaves (F2-H)	9.3	401.1 ± 24.7
	Stems (F2-T)	0.8	225.0 ± 12.5
Gema (GE)	Leaves (GE-H)	7.7	480.5 ± 5.2
	Stems (GE-T)	4.8	391.7 ± 4.2
Sarapiquí (S1)	Leaves (S1-H)	11.1	364.0 ± 6.3
	Stems (S1-T)	1.8	366.7 ± 22.2
	Bark (S1-C)	10.6	494.3 ± 2.8
	Wood (S1-M)	2.1	166.7 ± 9.5

^1^ g of extract/g of dry material expressed as %; ^2^ mg of gallic acid equivalent/g extract.

### 2.2. Phenolic Profile of U. tomentosa Extracts Determined by UPLC/TQ-ESI-MS

**Figure 1:**
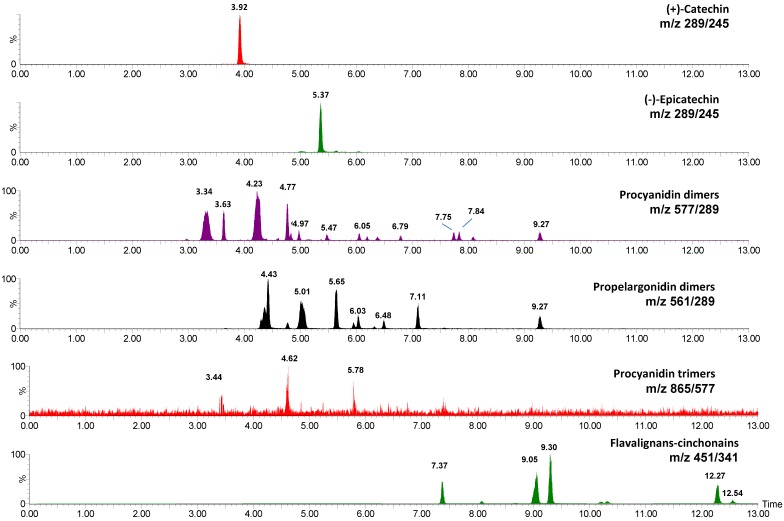
MRM chromatograms (UPLC-DAD-ESI-TQ-MS) for flavan-3-ols in *U. tomentosa* leave extracts.

UPLC/TQ-ESI-MS analysis was performed in the 22 extracts obtained from *U. tomentosa*, as described in the experimental section. A total of 32 phenolic compounds were determined, including hydroxybenzoic acids (benzoic, salicylic, 4-hydroxybenzoic, protocatechuic, gallic, vanillic and syringic acids), hydroxycinnamic acids (*p*-coumaric, caffeic, ferulic and isoferulic acids), flavan-3-ols (monomers, procyanidin dimers and trimers, and propelargonidin dimers) and flavalignans (cinchonains) (Table 2 and Table 3). Table S1 (Supplementary Materials) reports their MS/MS parameters and, as an example, Figure 1 shows the MRM (Multiple Reaction Monitoring) transitions for flavan-3-ols monomers [(+)-catechin and (−)-epicatechin, *m*/*z* 289/245], procyanidin dimers (*m*/*z* 577/289), propelargonidin dimers (*m*/*z* 561/289), procyanidin dimers (*m*/*z* 865/577) and cinchonains (*m*/*z* 451/341) found in an extract from *U. tomentosa* leaves. At least four different propelargonidin dimers (with retention times of 4.43, 5.01, 5.65 and 9.27 min) were detected in all the *U. tomentosa* extracts, with the only exceptions of the wood extracts from Asomat (AS-M) and Fortuna 1 (F1-M) (Table 3). To our knowledge, our results reveal for the first time the presence of propelargonidins in *U. tomentosa*.

As expected, some variability was observed in the content of phenolic compounds among the different origins/locations of the plants. Even with that, our results showed certain particularities in the phenolic composition of the different parts of *U. tomentosa* (bark, leaves, stems and wood). Extracts from leaves were particularly rich in procyanidin dimers (1250.5–8739.3 µg/g of extract) and propelargonidin dimers (4015.4–9722.4 µg/g), also with certain origin-dependent contributions of flavan-3-ol monomers (*i.e.*, F2-H, 4906.7 µg/g) and flavalignans (*i.e.*, S1-H, 6301.8 µg/g) (Table 2). Procyanidin dimers were also the most abundant phenolic group in bark extracts 1702.9–7746.1 µg/g), with important contributions of hydroxybenzoic acids (*i.e.*, P1-C, 2383.2 µg/g) and flavalignans (*i.e.*, S1-C, 2100.7 µg/g) (Table 2). Extracts from stems exhibited similar phenolic profile to bark extracts, procyanidin dimers (1551.4–10111.2 µg/g) being the most abundant group, followed by hydroxybenzoic acids (1122.7–4482.5µg/g) and flavalignans (718.9–2802.7 µg/g) (Table 3). For these three types of extracts (leaves, bark and stems), (−)-epichatechin was found in larger concentration than (+)-catechin. Among procyanidin dimers, B4 [(+)-(catechin-(4α→8)-(−)-epicatechin] was the most abundant procyanidin in the extracts of leaves, bark and stems, followed by B3 [(+)-catechin-(4α→8)-(+)-catechin] (especially in leaves extracts) and B2 [(−)-epicatechin-(4α→8)-(−)-epicatechin] (especially in bark and stems extracts) (Table 2 and Table 3). However, in the case of wood extracts, phenolic compounds mainly belonged to the hydroxybenzoic acids (747.9–1326.6 µg/g) and flavalignans (484.4–860.5µg/g) groups (Table 3), but their contents were relatively low in comparison to the other type of extracts. Finally, the sum of the contents of individual compounds (data not shown) ranged from external to internal plant parts in the following order: leaves > stem ~ bark > wood, and as seen for their total phenolic content (Table 1).

**Table 2:** Phenolic composition of extracts from bark and leaves of *U. tomentosa*.

Compounds	Bark Extracts			Leaves Extracts							
	AS	P1	S1	AS	F1	F2	GE	L1	L2	P1	S1
	Concentration (µg/g Extract)										
*Hydroxybenzoic acids*											
Benzoic acid	83.5 ± 3.7	1747.0 ± 20.4	134.5 ± 0.2	55.8 ± 5.3	20.6 ± 0.9	71.0 ± 3.1	nd	57.9 ± 5.6	44.1 ± 4.4	34.9 ± 3.5	50.2 ± 5
Salicylic acid	1.1 ± 0.1	78.7 ± 6.5	14.0 ± 0.6	12.0 ± 0.2	13.1 ± 0.2	21.7 ± 0.3	0.9 ± 0	11.9 ± 0.7	9.8 ± 0.5	21.7 ± 0.6	31.2 ± 3.1
4-Hydroxybenzoic acid	25.1 ± 1.2	74.1 ± 4.1	55.4 ± 2.9	38.2 ± 1.3	158.4 ± 3.5	430.3 ± 0.1	18.2 ± 1.3	15.8 ± 0.7	22.5 ± 1	47.3 ± 0.5	12.3 ± 0
Protocatechuic acid	168.0 ± 2.3	331.9 ± 24.1	79.4 ± 8.5	68.0 ± 1	254.1 ± 0.6	684.5 ± 15.7	18.1 ± 0.9	31.5 ± 0.9	32.6 ± 1.6	78.5 ± 0.2	21.5 ± 0.9
Gallic acid	9.4 ± 0.6	22.8 ± 1.1	14.5 ± 0.8	4.5 ± 0.3	15.5 ± 1.6	24.6 ± 1	6.7 ± 0.4	22.4 ± 0.7	3.4 ± 0	18.7 ± 1.2	9.0 ± 0.9
Vanillic acid	17.5 ± 1.7	104.1 ± 8.9	62.1 ± 3.3	nd	6.7 ± 0.7	10.5 ± 1.1	nd	3.8 ± 0.2	4.3 ± 0.4	5.1 ± 0.5	3.7 ± 0.37
Syringic acid	3.4 ± 0.2	24.6 ± 1.4	23.2 ± 1	nd	5.9 ± 0.6	6.9 ± 0.7	nd	3.4 ± 0.3	4.1 ± 0.3	2.8 ± 0.1	nd
∑ *Hydroxybenzoic acids*	309.8	2383.2	383.1	178.5	474.3	1249.5	43.9	146.4	120.8	209	127.9
*Hydroxycinnamic acids*											
*p*-Coumaric acid	0.9 ± 0.1	4.2 ± 0.4	9.5 ± 0.5	31.5 ± 1.4	37.1 ± 0.2	209.4 ± 1.7	11.2 ± 1	4.5 ± 0.4	5.1 ± 0.1	9.4 ± 0.1	3.5 ± 0.3
Caffeic acid	2.1 ± 0.1	12.5 ± 0.8	9.7 ± 1	6.2 ± 0	14.6 ± 0.2	35.4 ± 0.4	8.0 ± 0.3	5.5 ± 0.3	6.7 ± 0.3	4.1 ± 0.4	2.4 ± 0
Ferulic acid	nd	5.6 ± 0.4	6.6 ± 0.7	30.7 ± 1.5	29.9 ± 1	56.8 ± 2.7	13.5 ± 0.8	7.8 ± 0.5	15.5 ± 0.5	16.2 ± 1.4	11.5 ± 1.6
Isoferulic acid	nd	5.7 ± 0.4	2.2 ± 0.2	nd	10.5 ± 0.8	6.8 ± 0.7	nd	15.0 ± 0.9	nd	10.9 ± 0.2	13.1 ± 1.3
∑ *Hydroxycinnamic acids*	3.0	28.0	28.0	68.4	92.1	308.4	32.7	32.8	27.3	40.6	29.5
*Flavan-3-ols: monomers*											
(+)-Catechin	29.9 ± 2.8	238.4 ± 3.2	93.1 ± 9.3	569.1 ± 28.4	428.1 ± 3.1	2387.9 ± 152.3	202.6 ± 6.5	738.3 ± 36.4	203.4 ± 3	754.4 ± 0.4	1689.1 ± 48.8
(−)-Epicatechin	419.6 ± 9.6	1581.8 ± 87.2	678.2 ± 49.5	2062.3 ± 59.2	613.5 ± 17.2	2518.8 ± 34.7	189.9 ± 7.5	1054.7 ± 15	735.9 ± 23.9	963.9 ± 81.8	2128.0 ± 22.1
∑ *Monomers*	449.5	1820.2	771.3	2631.4	1041.6	4906.7	392.5	1793.0	939.3	1718.3	3817.1
*Flavan-3-ols: procyanidin dimers*											
Procyanidin B1	39.1 ± 1.7	170.6 ± 17.1	306.7 ± 30.7	105.9 ± 5.5	717.2 ± 11.2	454.6 ± 7.3	93.1 ± 6.1	1092.1 ± 83.7	474.4 ± 15.1	684.9 ± 15.2	612.1 ± 34.3
Procyanidin B2	827.7 ± 16.1	1651.8 ± 151.4	2184.9 ± 218.5	343.9 ± 1.4	887.3 ± 22	748.6 ± 0	128.2 ± 4.9	1254.9 ± 83.6	857.3 ± 44.7	930.2 ± 2.2	892.1 ± 48.9
Procyanidin B3	44.1 ± 4.1	128.3 ± 12.8	372.6 ± 37.3	96.3 ± 1.7	1518.3 ± 53.1	1562.3 ± 82.1	307.0 ± 6.6	2569.3 ± 142.5	623.9 ± 20.8	1686.6 ± 36.2	1869.2 ± 18.1
Procyanidin B4	661.6 ± 8.9	2175.1 ± 217.5	3962.1 ± 396.2	544.2 ± 28.8	2061.3 ± 66.9	2318.3 ± 64.4	602.7 ± 6.7	3089.5 ± 108	1852.6 ± 30.2	2630.7 ± 113.7	2593.2 ± 175.6
Procyanidin B5	130.4 ± 10.6	282.3 ± 26.8	563.4 ± 9.1	77.5 ± 5.6	180.2 ± 4.1	226.0 ± 15.9	0.0 ± 0	260.1 ± 26	143.5 ± 12	232.7 ± 7.8	351.9 ± 3.4
Procyanidin B7	nd	75.3 ± 7.5	108.4 ± 8.2	25.0 ± 0.4	185.4 ± 6.4	110.5 ± 11.1	45.0 ± 0.1	278.3 ± 3.6	99.8 ± 6	162.6 ± 9.5	140.3 ± 5.8
Procyanidin B (5.47 min)		101.0 ± 10.1	199.9 ± 14	28.4 ± 0.3	70.3 ± 2.4	94.7 ± 5.8	41.8 ± 2.1	157.8 ± 10.7	136.2 ± 5.3	106.8 ± 6.9	129.7 ± 12.4
Procyanidin B (9.27 min)		16.7 ± 0.5	48.1 ± 4.8	71.1 ± 3.9	40.0 ± 0.2	70.5 ± 6.4	32.7 ± 1.8	37.3 ± 3.7	26.5 ± 2.4	99.8 ± 0.7	227.7 ± 7.7
∑ *Procyanidin dimers*	1702.9	4601.1	7746.1	1292.3	5660.0	3272.6	1250.5	8739.3	4214.2	6534.3	6816.2
*Flavan-3-ols: properlargonidin dimers*											
Propelargonidin dimer (4.43 min)	23.4 ± 1	85.5 ± 3.7	308.9 ± 30.9	1181.4 ± 15.2	4235.8 ± 58.3	3691.4 ± 29.8	2767.6 ± 47.2	3598.9 ± 106.9	1688.7 ± 48.1	5021.9 ± 263.7	3669.9 ± 276.6
Propelargonidin dimer (5.01 min)	141.3 ± 11.7	319.4 ± 31.9	1493.6 ± 149.4	1442.5 ± 18.2	2498.9 ± 45.6	2675.8 ± 35.3	2227.9 ± 46.6	2421.8 ± 116.5	1328.6 ± 54.1	3147.7 ± 111.7	2633.1 ± 103.4
Propelargonidin dimer (5.65 min)	168.0 ± 4.7	0.1 ± 0.1	80.3 ± 5.2	1570.9 ± 46.7	428.3 ± 16.2	627.3 ± 2.4	995.7 ± 12.8	437.8 ± 2.8	1652.8 ± 16.2	654.9 ± 57.9	789.4 ± 25.8
Propelargonidin dimer (9.27 min)	23.8 ± 0.7	96.4 ± 9.6	375.8 ± 37.6	270.6 ± 18.1	662.1 ± 15.4	555.2 ± 5.6	252.1 ± 5.6	729.1 ± 54.2	283.9 ± 4.1	897.9 ± 15.5	695.5 ± 31.6
∑ *Properlargonidin dimers*	365.5	501.4	2258.6	4465.4	7825.1	7549.7	6243.3	4036.7	4954	9722.4	7787.9
*Flavan-3-ols: procianidin trimers*											
Trimer T2	nd	0.9 ± 1.3	nd	nd	nd	nd	nd	8.1 ± 8.8	nd	nd	5.9 ± 8.4
Procyanidin C1	436.9 ± 45.5	133.7 ± 8.7	211.3 ± 21.1	nd	51.3 ± 5.1	29.7 ± 3.0	63.1 ± 6.3	46.5 ± 4.6	139.5 ± 2.6	22.2 ± 1.4	16.0 ± 0.9
Trimer B (4.62 min)	nd	23.2 ± 0.9	83.3 ± 34.3	nd	56.0 ± 3.8	63.7 ± 6.4	84.6 ± 5.3	101.6 ± 8.1	273.3 ± 8.6	53.7 ± 0.1	62.3 ± 6.2
∑ *Procyanidin trimers*	436.9	157.8	294.6	nd	107.3	93.4	147.7	156.2	412.8	75.9	84.2
*Flavalignans*											
Cinchonain (7.37 min)	149.7 ± 4.4	584.1 ± 53.8	937.8 ± 40.9	582.0 ± 2.1	709.5 ± 10.6	422.3 ± 18.8	177.1 ± 3.7	544.8 ± 41.3	537.3 ± 15.7	84.1 ± 8.4	1009.2 ± 95.9
Cinchonain (9.05 min)	12.5 ± 0.8	49.8 ± 5	129.1 ± 0	517.2 ± 23.1	1145.9 ± 53.3	896.3 ± 25.1	437.6 ± 9.8	973.8 ± 39.5	771.1 ± 26.1	108.0 ± 1	1939.9 ± 89.1
Cinchonain (9.30 min)	13.1 ± 0.9	50.0 ± 5	129.7 ± 11.4	843.0 ± 2.3	1152.2 ± 15.8	1040.2 ± 54	645.3 ± 13.5	1215.4 ± 73.4	915.3 ± 28.9	103.6 ± 7.9	2276.7 ± 82.9
Cinchonain (12.27 min)	189.9 ± 4.5	541.3 ± 90.1	904.1 ± 154.1	770.0 ± 25.8	545.0 ± 6.5	542.4 ± 3.5	255.8 ± 14	480.4 ± 0.8	540.5 ± 23.6	59.1 ± 6.1	1076.0 ± 5.8
∑ *Flavalignans*	365.2	1225.2	2100.7	2712.2	3552.6	2901.2	1515.8	3214.4	2764.2	354.8	6301.8

AS—Asomat; L1—Los Chiles 1; L2—Los Chiles 2; Palacios—P1; F1—Fortuna 1; F2—Fortuna 2; GE—Gema; S1—Sarapiquí; nd—not detected.

**Table 3:** Phenolic composition of extracts from stems and wood of *U. tomentosa*.

Compounds	Stems							Wood			
	AS	F1	F2	GE	L1	P1	S1	AS	F1	P1	S1
	Concentration (µg/g Extract)										
*Hydroxybenzoic acids*											
Benzoic acid	130.6 ± 4.8	989.1 ± 51.3	498.2 ± 38.3	14.0 ± 1	342.4 ± 12	506.0 ± 2.1	365.6 ± 21.5	1188.3 ± 87.8	23.1 ± 2.3	1718.5 ± 43.8	1285.7 ± 119.9
Salicylic acid	47.1 ± 1.4	75.5 ± 1.5	185.3 ± 2.4	39.1 ± 1.5	70.5 ± 4	95.2 ± 0.2	101.5 ± 0.9	37.8 ± 2.6	34.5 ± 2.3	53.1 ± 0.1	58.2 ± 1.4
4-hydroxybenzoic acid	19.2 ± 0.9	160.2 ± 3.3	292.6 ± 15.4	23.1 ± 0.8	46.0 ± 2	81.5 ± 2	74.2 ± 5.7	115.6 ± 3.6	42.3 ± 0.7	38.0 ± 0.9	139.2 ± 2.4
Protocatechuic acid	126.6 ± 2.8	777.5 ± 15.3	1313.7 ± 54.6	114.2 ± 7.3	249.8 ± 11.5	276.0 ± 1.7	148.6 ± 7.6	181.2 ± 3.8	464.4 ± 10.1	107.2 ± 1.9	120.2 ± 0.8
Gallic acid	25.6 ± 1.6	33.5 ± 0.2	64.2 ± 1	29.1 ± 3	37.5 ± 0.8	47.3 ± 1.5	30.9 ± 1.2	17.1 ± 1.5	27.7 ± 0.2	27.2 ± 0.7	22.3 ± 2
Vanillic acid	85.5 ± 4.4	467.7 ± 2.8	676.1 ± 52.7	104.9 ± 10.4	241.8 ± 11.5	334.6 ± 16.9	238.4 ± 1.9	214.0 ± 5.8	77.6 ± 1.2	152.3 ± 0.5	322.7 ± 3.4
Syringic acid	25.8 ± 2.3	157.2 ± 11	190.8 ± 19.1	22.8 ± 1.6	65.3 ± 0.4	78.2 ± 6.8	79.8 ± 4	76.5 ± 7.8	39.9 ± 0.8	69.9 ± 3.3	160.3 ± 5.7
∑ *Hydroxybenzoic acids*	4482.5	1999.2	2276.1	4322.7	1122.7	1669.6	3246.1	1326.6	747.9	1296.7	1073.1
*Hydroxycinnamic acids*											
p-Coumaric acid	7.0 ± 0.3	17.1 ± 1.5	107.4 ± 1.6	10.6 ± 0.7	7.8 ± 0.5	11.3 ± 0.3	17.9 ± 1.5	22.6 ± 1.9	11.3 ± 1.1	6.6 ± 0.6	31.7 ± 2
Caffeic acid	10.4 ± 0.5	29.7 ± 1.9	57.3 ± 1	8.5 ± 0.6	12.3 ± 0.4	18.7 ± 0.2	18.4 ± 1	42.2 ± 0.7	122.8 ± 9.6	34.3 ± 3	44.4 ± 0.3
Ferulic acid	12.5 ± 1.3	43.9 ± 4	148.4 ± 14.8	21.5 ± 1.4	18.1 ± 0.5	24.2 ± 0.1	27.8 ± 1.2	45.0 ± 3.3	14.4 ± 1.4	30.3 ± 3	28.2 ± 2.3
Isoferulic acid	nd	17.9 ± 1.8	24.5 ± 1.9	11.6 ± 1	25.0 ± 2.4	20.1 ± 2	29.3 ± 2.9	39.3 ± 2.4	12.6 ± 1.3	16.1 ± 1.6	21.7 ± 2.2
∑*Hydroxycinnamic acids*	29.9	108.6	337.6	51.6	63.2	74.3	93.4	149.1	161.1	87.3	126.0
*Flavan-3-ols: monomers*											
(+)-Catechin	124.7 ± 11.7	92.1 ± 5.8	394.7 ± 7.3	623.7 ± 17.7	211.3 ± 4.5	219.2 ± 11.6	201.2 ± 0.9	nd	57.3 ± 5.7	9.8 ± 0.7	20.5 ± 3.9
(−)-Epicatechin	1106.7 ± 35	337.2 ± 1.7	758.4 ± 56.3	1964.8 ± 148.4	847.6 ± 0.2	815.3 ± 1.1	1000.5 ± 6.5	19.5 ± 0.4	167.1 ± 5.7	62.1 ± 3.7	127.4 ± 2
∑ *Monomers*	1231.4	429.3	1153.1	2588.5	1058.9	1034.5	1201.7	19.5	224.4	71.9	147.9
*Flavan-3-ols: procyanidin dimers*											
Procyanidin B1	317.3 ± 6.6	116.0 ± 11.1	21.6 ± 1.7	433.7 ± 31.3	243.8 ± 6	343.3 ± 11.2	404.2 ± 11.8	nd	nd	nd	21.1 ± 0.6
Procyanidin B2	2049.6 ± 20.6	629.7 ± 14.1	157.4 ± 7.8	1644.7 ± 141.4	949.5 ± 95	1166.9 ± 1.3	1867.7 ± 96.1	nd	14.8 ± 1.5	19.7 ± 1.1	363.9 ± 31.9
Procyanidin B3	842.3 ± 4.6	440.9 ± 4.8	226.3 ± 1.3	1746.8 ± 2.4	658.1 ± 11.3	1145.1 ± 12.8	1080.0 ± 4.1	nd	2.4 ± 0.2	0.1 ± 0	55.3 ± 5.5
Procyanidin B4	5500.0 ± 45.6	1861.2 ± 68.4	1062.9 ± 106.3	5443.4 ± 338.9	2360.8 ± 192.1	3569.1 ± 92.1	4152.2 ± 117.7	nd	35.9 ± 1.5	29.7 ± 0.3	554.2 ± 18.2
Procyanidin B5	446.9 ± 12.5	93.9 ± 5.1	45.8 ± 4.6	382.2 ± 37	177.8 ± 17.8	202.8 ± 18.1	522.9 ± 52.3	nd	4.1 ± 0.4	5.5 ± 0.5	54.7 ± 4.7
Procyanidin B7	158.4 ± 6.5	48.8 ± 0.9	20.0 ± 2	204.7 ± 1.4	80.9 ± 3.4	122.7 ± 0.2	163.2 ± 16.3	nd	nd	nd	15.7 ± 1.2
Procyanidin B (5.47 min)	195.0 ± 15.9	34.9 ± 3.1	17.4 ± 1.7	200.1 ± 15.6	81.2 ± 8.1	113.4 ± 8.2	178.6 ± 17.9	nd	nd	nd	15.9 ± 1.5
Procyanidin B (9.27 min)	31.6 ± 2.3	nd	nd	55.6 ± 1.7	13.3 ± 1.2	nd	40.9 ± 4.1	nd	nd	nd	5.6 ± 0.6
∑ *Procyanidin dimers*	9541.1	3225.4	1551.4	10111.2	4565.4	6663.3	8409.7		57.2	55.0	722.5
*Flavan-3-ols: propelargonidin dimers*											
Propelargonidin dimer (4.43 min)	416.2 ± 11.8	462.0 ± 4.5	127.1 ± 8.5	724.6 ± 50.9	250.9 ± 17.6	773.3 ± 25.5	610.1 ± 4.2	nd	nd	0.1 ± 0	21.3 ± 2.1
Propelargonidin dimer (5.01 min)	1880.3 ± 31.3	939.5 ± 16.3	452.9 ± 45.3	1577.4 ± 117.7	637.1 ± 63.7	1799.6 ± 76.6	1892.5 ± 100.4	nd	13.0 ± 0.5	7.0 ± 0.7	110.0 ± 10.2
Propelargonidin dimer (5.65 min)	963.0 ± 21.5	45.9 ± 4.6	26.4 ± 2.6	737.1 ± 71.4	43.6 ± 4.4	104.5 ± 9	115.7 ± 5.2	nd	0.1 ± 0	0.1 ± 0	0.1 ± 0
Propelargonidin dimer (9.27 min)	208.8 ± 2.3	90.9 ± 9.1	49.2 ± 4.9	158.7 ± 15.7	69.2 ± 6.9	209.4 ± 20.9	285.7 ± 14.7	nd	nd	3.8 ± 0.4	24.4 ± 2.4
∑ *Propelargonidin dimers*	3468.3	1538.3	655.6	3197.8	1000.8	2886.8	2904.0		13.1	11.0	155.8
*Flavan-3-ols: procyanidin trimers*											
Trimer T2	104.3 ± 10	nd	nd	108.7 ± 9.1	5.4 ± 5.1	4.6 ± 4.1	13.9 ± 0.8	nd	nd	nd	nd
Procyanidin C1	1095.0 ± 41.2	60.2 ± 6	nd	884.7 ± 61.6	116.4 ± 11.6	83.6 ± 4.4	226.4 ± 22.6	nd	nd	nd	33.8 ± 3.4
Trimer B (4.62 min)	987.0 ± 39.7	38.6 ± 3.9	1.7 ± 0.1	1000.7 ± 58.6	134.8 ± 13.5	113.4 ± 7.5	221.2 ± 9.6	nd	1.8 ± 0.1	1.7 ± 0.1	1.8 ± 0
∑ *Procyanidin trimers*	2186.3	98.8	1.7	1994.1	256.6	201.6	461.5		1.8	1.7	35.6
*Flavalignans*											
Cinchonain (7.37 min)	1122.9 ± 17.8	591.4 ± 21.7	385.7 ± 38.6	608.7 ± 66.1	315.1 ± 30.7	483.6 ± 2.4	830.5 ± 25.4	394.4 ± 3.6	220.9 ± 6.5	400.9 ± 6	339.5 ± 4.2
Cinchonain (9.05 min)	339.8 ± 4	150.9 ± 0.9	281.7 ± 27.3	667.0 ± 62.8	93.6 ± 7.4	139.3 ± 13	278.2 ± 27.8	18.2 ± 0.4	29.4 ± 2.9	30.7 ± 3.1	20.6 ± 1.1
Cinchonain (9.30 min)	329.7 ± 6.6	130.4 ± 4.2	270.4 ± 8.8	632.5 ± 56.2	88.4 ± 7.6	114.4 ± 4.2	224.9 ± 6.8	27.6 ± 1.5	22.8 ± 0.9	26.8 ± 0.1	25.0 ± 2.5
Cinchonain (12.27 min)	1010.3 ± 7.1	422.6 ± 7.9	393.1 ± 19.5	557.5 ± 29	221.8 ± 17	339.3 ± 7.6	704.7 ± 27.4	420.3 ± 14.4	211.3 ± 0.3	390.4 ± 20.7	321.2 ± 11.3
∑ *Flavalignans*	2802.7	1295.3	1330.9	2465.7	718.9	1076.6	2038.3	860.5	484.4	848.8	706.3

AS—Asomat; L1—Los Chiles 1; L2—Los Chiles 2; Palacios—P1; F1—Fortuna 1; F2—Fortuna 2; GE—Gema; S1—Sarapiquí; nd—not detected.

**Figure 2:**
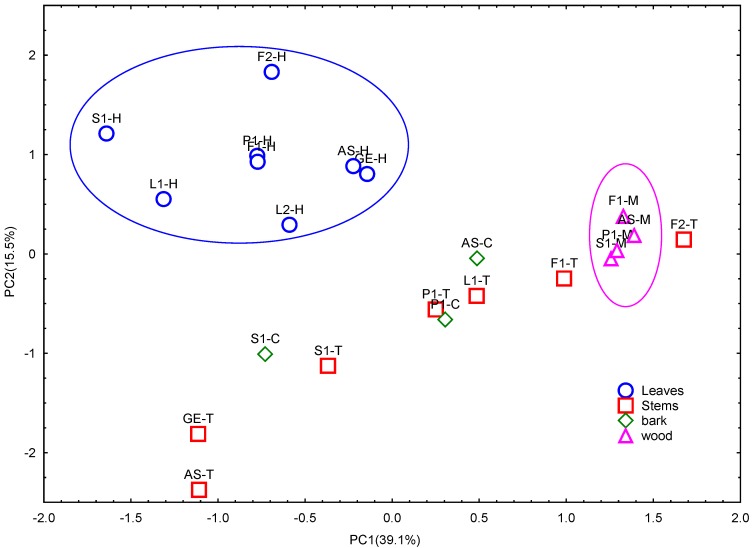
Plane defined by the two first principal components (PC1 and PC2) resulting from the PCA analysis of total phenolic content (PT, PRO) and individualized phenolic composition of *U. tomentosa* phenolic extracts (*n* = 22).

To summarize the results from these analyses, a statistical Principal Component Analysis (PCA) was performed for the 22 extracts taking into consideration 33 variables (individual phenolic compounds + total phenolic content) (Figure 2). Two components (PC1 and PC2) were obtained: PC1 represented 39% of variance and showed a negative correlation (loadings > 0.7) with the total phenolic content, procyanidins B1, B3, B7, and with an unknown procyanidin (RT = 5.47 min), as wells as positive correlation with syringic acid. PC2 represented 16% of variance and was negatively correlated with procyanidin C1. In the plane represented by the two components, leaves extracts were grouped separately from the rest of the samples. Wood extract presented the highest values in PC1, and were also separated in another homogeneous group. On the other hand, bark and stems showed a high variability and overlapping in the planes of both components, indicating that phenolic composition of these parts can be strongly affected by geographical origin or location.

### 2.3. ^13^C-NMR Analysis of U. tomentosa Extracts

**Figure 3:**
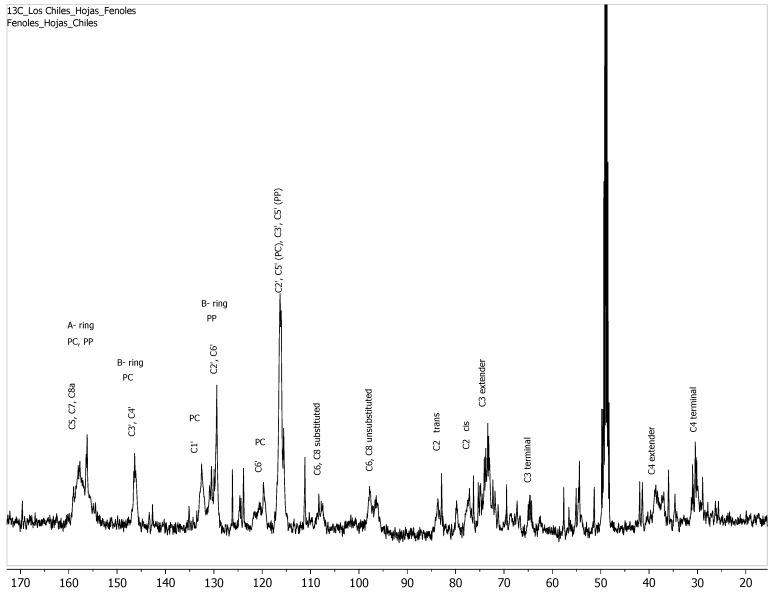
^13^C-NMR (MeOD) for a polyphenolic extract of leaves (L1-H) from *U. tomentosa.*

**Figure 4:**
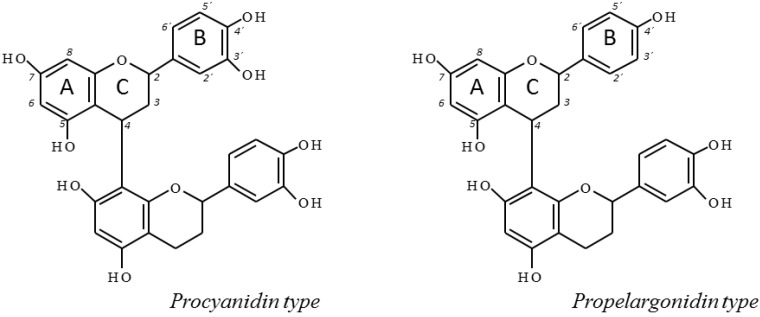
Procyanidin and propelargonidin general chemical structures.

As in agreement with the UPLC-MS/MS results, the ^13^C-NMR analysis of *U. tomentosa* extracts showed characteristic signals for procyanidins and propelargonidins, respectively reported by Czochanska [15] and Fu [16] for other substrates. As an example, Figure 3 illustrates the ^13^C-NMR spectra of an extract from *U. tomentosa* leaves. Signals in the region between δ 160 and 150 ppm corresponded to carbons C5, C7 and C8 of the A ring from both procyanidins and propelargonidins (Figure 4). Distinctive signals characteristic of procyanidins were found at δ 145 ppm corresponding to C3′ and C4′ of the B ring and at δ 132.6 ppm corresponding to C1′. In addition, signals characteristic of propelargonidins were found at δ 130.1 ppm attributed to C1′ and at δ 129.4 ppm corresponding to carbons C2′ and C6′ of the B ring. Other characteristic signals appear at δ 119 ppm (C6′) for procyanidins and the cluster of peaks between δ 115 and 117 ppm that correspond to C2′, C5′ (procyanidins) and C3′, C5′ (propelargonidins). The signals between δ 95 and 96 ppm are assigned to C6 and C8 from the A ring, when this unit does not have a substituent. Furthermore, the signal at δ 108 ppm correspond to C6 and C8 when the A ring is bonded to another monomer unit through C4→C8 and C4→C6 type bonds, in agreement with the different isomers detected by UPLC-MS/MS analysis. On the other hand, the region between δ 90 and 70 ppm is useful to determine the stereochemistry of the C ring. In fact, two sets of signals are found at δ 83 and 76 ppm for C2 indicating the presence of both isomers, 2,3-*trans* and 2,3-*cis*, respectively, for the two different type of proanthocyanidins, corresponding to catechin and epicatechin units (procyanidins) and afzelechin and epiafzelechin units (propelargonidins). Finally, the broad signal between δ 74 and 73 ppm correspond to C3 extended units (both for isomers *cis* and *trans*) while peaks between δ 65 and 64 ppm correspond to C3 terminal units; and C4 corresponding to extended units for both *cis* and *trans* isomers appear as a broad peak between δ 38 and 36 ppm while C4 from terminal units showed several signals in the region between δ 29 and 27 ppm (Figure 3).

**Figure 5:**
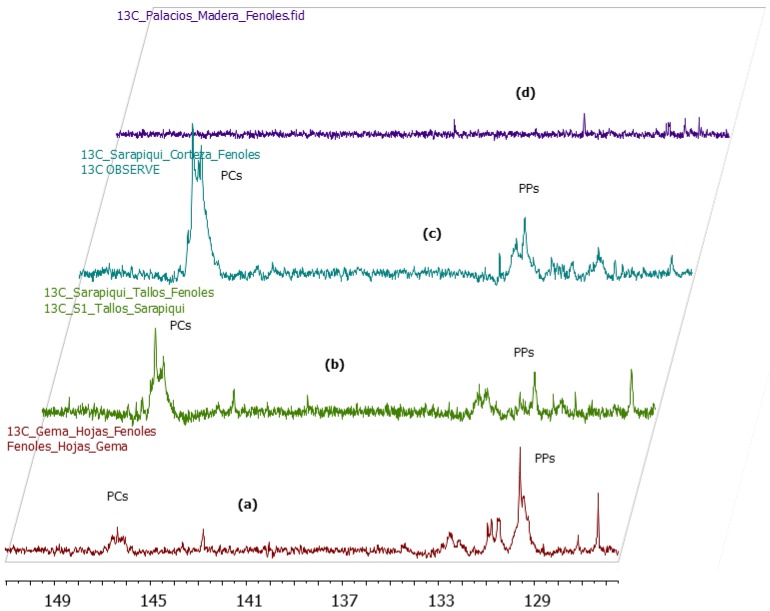
^13^C-NMR of leaves, stems, bark and wood extracts of *U. tomentosa:* (**a**) leaves (GE-H); (**b**) stems (S1-T); (**c**) bark (S1-C) and (**d**) wood (P1-M). Signals corresponding to procyanidins (PCs) and propelargonidins (PPS) are indicated.

In addition, ^13^C-NMR spectra allowed comparison of the relative abundance of procyanidins and propelargonidins. Figure 5 shows amplifications of ^13^C-NMR spectra for the four different parts of *U. tomentosa*. Spectra corresponding to leaves extracts clearly showed stronger signals at δ 129–130 ppm corresponding to C2′, C6′ and C1′ of propelargonidins in respect to the signal at δ 145–146 ppm characteristic of C2′ and C3′ of procyanidins, which was in agreement with the results from the UPLC-MS/MS (Table 2). Extracts from stems and bark samples had less preponderance of propelargonidins in respect to procyanidins (Figure 5b,c, respectively). Finally, the spectra corresponding to *U. tomentosa* wood (Figure 5d, P1-M) showed almost no significant signals in the studied region, which was also in agreement with the results from the UPLC-MS/MS (Table 3).

Our study proved the usefulness of both UPLC-DAD-ESI-TQ MS and ^13^C-NMR techniques in the characterization of the phenolic composition of *U. tomentosa*. One relevant contribution of this study was the identification of four propelargonidin dimers, although their complete structures remain to be elucidated. In addition, it has clearly showed the particularities of the different parts (leaves, stems, bark and wood) of the *U. tomentosa* plant concerning their phenolic content and profile. All of these add new valuable information to the existing literature concerning this topic [8,9,10,11,12,13].

### 2.4. Alkaloids Contents of U. tomentosa Extracts

**Figure 6:**
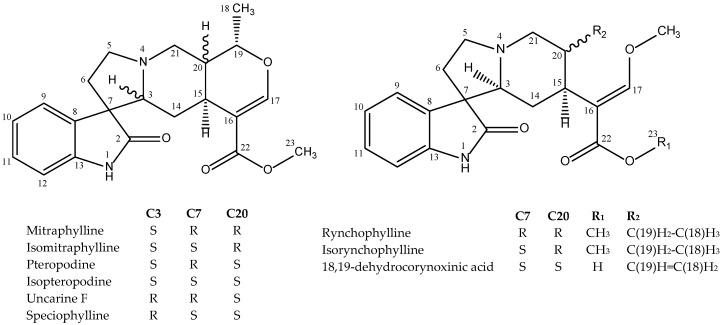
Tetracyclic and pentacyclic oxindole alkaloids identified in *U. tomentosa* extracts.

Although the main objective of the research was to study in detail the composition of phenolic extracts of *U. tomentosa*, alkaloids-rich extracts were obtained through acid-base extraction from the plant residue after phenolic extraction (Section 3.3). The extraction yields for the alkaloid extracts were acceptable, ranging from to 0.44% to 0.81% for leaves and from 0.20% to 0.35% for bark (Table 4). Following the experimental protocol described in Section 3.7, six known pentacyclic (mitraphylline, isomitraphylline, uncarine F, pteropodine, isopteropodine and speciophylline) and two known tetracyclic (rhynchophylline and isorhynchophylline) alkaloids (Figure 6) were identified through NMR data obtained (Figures S1–S10, Supplementary Materials) and quantified through HPLC in extracts of leaves and bark (Table 4), in agreement with data reported in the literature [2]. The tetracyclic alkaloids (rhynchophylline and isorhynchophylline) were present in almost all the extracts, reaching isorhynchophylline the highest value (509 ± 1 mg/100 g of dry material) in the leaf extract from the location of Palacios (P1-H). For the same plant location, the extracts from leaves showed higher content of rhynchophylline and isorhynchophylline than the extracts from bark (Table 4). On the contrary, the pentacyclic alkaloids mitraphylline, isomitraphylline, pteropodine and isopteropodine tended to be more abundant in the extracts from bark in comparison to leaf extracts, with the exception of the location of Los Chiles (L1-H) for mitraphylline and isomitraphylline, and the location of Asomat (AS-H) for pteropodine and isopteropodine (Table 4). Finally, speciophylline, uncarine F were only present in the bark extract from the location of Asomat (AS-C) (Table 4). Total alkaloid content in the extracts obtained from the different locations ranged from 95.0 to 275 mg/100 g of dry material for bark extracts, and from 330 to 704 mg/100 g of dry material for leaves extracts. For the same location, the leaf extracts were between two- and seven-fold alkaloid-richer than the bark extracts (Table 4). In addition, a minor alkaloid was isolated and identified as 18,19-dehydrocorynoxinoic acid when comparing NMR analyses (Figures S11 and S12, Supplementary Materials) with data published by Yuan [17].

Above all, this study confirmed the applicability of subsequent extractions of the plant material to obtain separate phenolic- and alkaloid-rich extracts in a more rational and environmental-friendly way [18].

## 3. Experimental Section

### 3.1. Plant Material

*Uncaria tomentosa* samples were collected from different places in Costa Rica, some of them from local communities that grow the plant, namely Asomat (AS), AromaTica-Fortuna (F1 and F2) and Gema (GE) in the northern part of the country as well as Aprolece-Palacios (P1) in the Caribbean; and others growing in the wild in Los Chiles (L1 and L2) in the northern part of Costa Rica and Sarapiqui (S1) in the Caribbean. Vouchers for all plants are deposited in the Costa Rican National Herbarium, under series no. AQ2953, AQ2959, AQ2968, AQ3246, AQ3331, AQ3332, AQ3508 and AQ3510, respectively. The plant material was separated in its different parts (leaves, stem, bark and wood) and then dried in a stove at 40 °C, being turned over every 24 h for a week until totally dry. The dried material was then ground and preserved in plastic recipients.

**Table 4:** Extraction yield and alkaloid content of extracts from *U. tomentosa*.

Sample	Extraction Yield (%) ^1^	Alkaloid Content (mg/100 g Dry Plant Material) ^2^								
		S	R	M	UF	IR	IM	P	IP	∑ Alkaloids
AS-C	0.20	16.8 ± 0.2	nd	13.6 ± 0.1 ^a^	10.1 ± 0.1	nd	7.92 ± 0.15 ^a^	50.2 ± 0.4 ^a^	36.0 ± 0.3 ^a^	135
AS-H	0.44	nd	85.1 ± 3.9 ^a^	nd	nd	185 ± 3 ^a^	nd	64.3 ± 1.3 ^b^	68.9 ± 1.4 ^b^	403
F1-H	0.81	nd	221 ± 2 ^b^	nd	nd	483 ± 5 ^b^	nd	nd	nd	704
GE-H	0.79	nd	195 ± 1 ^c^	nd	nd	350 ± 2 ^c^	nd	nd	nd	545
L1-C	0.32	nd	nd	9.22 ± 0.15 ^b^	nd	nd	26.9 ± 0.1 ^b^	54.9 ± 0.1 ^c^	55.3 ± 0.1 ^c^	146
L1-H	0.78	nd	16.6 ± 0.3 ^d^	135 ± 1c	nd	nd	179 ± 1 ^c^	nd	nd	330
P1-C	0.23	nd	27.3 ± 0.4 ^e^	nd	nd	57.4 ± 0.1 ^d^	nd	4.72 ± 0.25 ^d^	5.65 ± 0.29 ^d^	95.0
P1-H	0.72	nd	190 ± 1 ^c^	nd	nd	509 ± 1 ^e^	nd	nd	nd	699
S1-C	0.35	nd	77.5 ± 0.1 ^f^	7.22 ± 0.95 ^d^	nd	177 ± 1 ^a^	6.42 ± 0.05 ^d^	nd	6.95 ± 0.19 ^d^	275
S1-H	0.65	nd	139 ± 1 ^g^	nd	nd	413 ± 3 ^f^	nd	nd	nd	552

^1^ g of extract/g of dry material expressed as %. S—speciophylline, R—rhynchophylline, M—mitraphylline, UF—uncarine F, IR—isorhynchophylline, IM—isomitraphylline, P—pteropodine, IP—isopteropodine; AS-C Asomat-bark sample; AS-H Asomat-leaves sample; F1-H Fortuna1-leaves sample; GE-H Gema-leaves sample; L1-C Los Chiles1-bark sample; L1-H Los Chiles1-leaves sample; P1-C Palacios-bark sample; P1-H Palacios-leaves sample; S1-C Sarapiquí-bark sample; S1-H Sarapiquí-leaves sample. nd—not detected. ^2^ Different letters in the same column indicate differences are significant at *p* < 0.05.

### 3.2. Extraction of Phenolic Compounds from the Different Parts of *U. tomentosa*

The dried material from each part of the plant (leaves, stem, bark and wood) was first extracted (0.05 g/mL) in a mixture of methyl ter-butyl ether (MTBE) and methanol (MeOH) 90:10 (*v*/*v*) at 25 °C during 30 min in ultrasound; subsequently, it was left standing for 24 h to obtain a non-polar extract of the samples. The solvent was removed by filtration and the extraction process was repeated once. The extracts were concentrated to dryness and washed with MeOH to extract residual polyphenols. After the non-polar extraction, the residual material (0.05 g/mL) was extracted with MeOH at 25 °C during 30 min in ultrasound and then it was left standing for 24 h. The solvent was removed by filtration and the extraction process was repeated twice. These three methanol extracts were combined with the MeOH washings of the dried non-polar extracts. MeOH was evaporated in a rotavapor at less than 40 °C, and the dried extract was then washed with hexane, MTBE and chloroform successively to deliver a polyphenolic rich-extract.

### 3.3. Obtention of Alkaloids Extracts

After the phenolic extraction, NH_3_ (20% *v*/*v* in MeOH) was added to the residual material and left at 25 °C during 30 min in ultrasound and subsequently it was left standing for 24 h. The solvent was removed by filtration. Afterwards, extraction with MTBE was performed at 25 °C during 30 min in ultrasound and then it was left standing for 24 h. The solvent was removed by filtration and the MTBE extraction process was repeated once. The extracts were combined and a solution of aqueous tartaric acid (20% *v*/*v*) was used three times to extract the alkaloids from the organic solvent. Next, the acidic phase was treated with a saturated solution of sodium carbonate to attain pH = 10, filtrated and the basic solution was extracted three times with MTBE. The organic solvent was then dried with anhydrous sodium sulfate, filtrated and concentrated to dryness in a rotavapor at less than 40 °C, to deliver an alkaloid rich-extract.

### 3.4. Determination of Total Phenolic Content

Total polyphenols were determined by a modification of the Singleton and Rossi method [19], which is based on the oxidation of the hydroxyl groups of phenols in basic media by the Folin-Ciocalteu (FC) reagent (mixture of phosphotungstic and phosphomolybdic acids of yellow colour). Briefly, each extract sample was dissolved in MeOH (0.1% HCl) and mixed with 0.5 mL of FC reagent. Then, 10 mL of Na2CO3 (7.5%) and water were added to complete 25 mL. A blank was prepared similarly but with 0.5 mL of MeOH (0.1% HCl) instead of the sample. After standing in the dark for 1 h, absorbance was measured at 750 nm and absorbance values were extrapolated in a gallic acid calibration curve. Values of total phenolic content were expressed as mg gallic acid equivalents (GAE)/g sample. Analyses were performed in triplicate.

### 3.5. Analysis of Phenolic Compounds by UPLC-DAD-ESI-TQ MS

An UPLC system coupled to an Acquity PDA eλ photodiode array detector (DAD) and an Acquity TQD tandem quadrupole mass spectrometer equipped with Z-spray electrospray interfece (UPLC-DAD-ESI-TQ MS) (Waters, Milford, MA, USA) was used to analyze the phenolic composition of *U. tomentosa* extracts. A solution of 5 mg/mL of the different phenolic extracts were prepared in acetonitrile:H_2_O (1:4). Separation (2 μL) was performed on a Waters^®^ BEH C18 column (2.1 × 100 mm; 1.7 μm) at 40 °C. A gradient composed of solvent A- water:acetic acid (98:2, *v*/*v*) and B-acetonitrile:acetic acid (98:2, *v*/*v*) was applied at flow rate of 0.5 mL/min as follows (21): 0–1.5 min: 0.1% B, 1.5–11.17 min: 0.1%–16.3% B, 11.17–11.5 min: 16.3%–18.4% B, 11.5–14 min: 18.4% B, 14–14.1 min: 18.4%–99.9% B, 14.1–15.5 min: 99.9% B, 15.5–15.6 min: 0.1% B, 15.6–18 min: 0.1% B. The DAD was operated in the 250–420 nm wavelength range at a 20 point/s rate and 1.2 nm resolution. The ESI parameters were: Capillary voltage, 3 kV; source temperature, 130 °C; desolvation temperature, 400 °C; desolvation gas (N_2_) flow rate, 750 L/h; cone gas (N_2_) flow rate, 60 L/h. The ESI was operated in negative mode. For quantification purposes, data were collected in the multiple reaction monitoring (MRM) mode, tracking the transition of parent and product ions specific for each compound, and using external calibration curves. For instance, transitions *m*/*z* 289/245 (monomers of flavan-3-ol, (+)-catechin and (−)-epicatechin), 577/289 (procyanidin dimers), *m*/*z* 561/289 (propelargonidin dimers), *m*/*z* 865/577 (procyanidin trimers), and *m*/*z* 451/341 (flavanolignans of cinchonain type). Commercial standards used were (+)-catechin, (−)-epicatechin, procyanidin B1 [(−)-epicatechin-(4β → 8)-(+)-catechin], procyanidin B2 [(−)-epicatechin-(4β → 8)-(−)-epicatechin], and procyanidin C1 [(−)-epicatechin-(4β → 8)-(−)-epicatechin-(4β → 8)-(−)-epicatechin] for optimizations, mass detector parameters and calibration curves. Assignment of other structures such as procyanidins B3 [(+)-catechin-(4α → 8)-(+)-catechin], B5 [(−)-epicatechin-(4β → 6)-(−)-epicatechin], B7 [(−)-epicatechin-(4β → 6)-(+)-catechin], and procyanidin trimer T2 [(−)-epicatechin-(4β → 8)-(−)-epicatechin- (4β → 8)-(+)-catechin], was carried out with standards isolated from other plants and confirmation by MS/MS spectrum. Due to the absence of commercial standards for propelargonidins and flavanolignans, an MS/MS spectrum was performed on the molecular ions at *m*/*z* 561 and *m*/*z* 451 to confirm the structure of both types of compounds, respectively. Quantification of flavalignans and propelargonidins was performed on the calibration curve of procyanidin B1 and (−)-epicatechin, respectively, while quantification of procyanidin B2 and B3 was carried out on the calibration curve of procyanidin B1. The limit of detection (LOD) and limit of quantification (LOQ) of the standards used are published elsewhere [20,21]. Analyses were performed in duplicate.

### 3.6. Analysis by ^13^C-NMR

^13^C nuclear magnetic resonance (NMR) spectra of the samples (25 mg) were recorded in CD_3_OD (0.75 mL) on a Bruker Ascend 400 MHz instrument.

### 3.7. Alkaloid Analysis

Pentacyclic alkaloids mitraphylline (M), isomitraphylline (IM), uncarine F (UF), pteropodine (P) and isopteropodine (IP) and speciophylline (S), and tetracyclic alkaloids rhynchophylline (R) and isorhynchophylline (IR) were identified in extracts and characterized by their ^1^H- and ^13^C-NMR spectra, as previously published in the literature [22,23]. An Agilent 1260 HPLC-DAD system with Chem-32 software was used. Analyses were performed using a Phenomenex Synergi Polar RP-80 column, 150 mm × 4.5 µm; a pre-column Phenomenex 15 mm × 4.5 µm and a solvent system consisting of A: MeOH, B: CH_3_CN and C: NH_4_OAc buffer (pH 5.0) with a linear gradient of A:B:C from 20:20:60 up to 35:35:30 in 35 min at flow of 1 mL/min, with detection done at 245 nm. Calibration curves of rhynchophylline and isopteropodine in a concentration range from 8–350 mg/L (R_2_ = 0.999, for both) were used for quantification of tetracyclic and pentacyclic oxyindole alkaloids, respectively. Analyses were run in triplicate.

For preparative separations of alkaloids, the same Agilent HPLC-DAD equipment was used with a semi-preparative Synergi Polar RP-80 column (150 mm × 10 um), a pre-column Phenomenex 15 mm × 10 µm and a solvent system consisting of A: MeOH, B: CH_3_CN and C: NH_4_OAc buffer (pH 5.0) with a linear gradient of A:B:C from 20:20:60 up to 35:35:30 in 35 min at flow of 1 mL/min. NMR analysis (Supplementary material) were performed on samples dissolved in CDCl_3_ in a Brucker Ascend 400 MHz instrument.

### 3.8. Statistical Analysis

Principal component analysis (PCA), from standardized variables, was applied to summarize the phenolic data from leaves, stem, bark and wood extracts from the U. tomentosa plant parts using the STATISTICA program for Windows, version 7.1 (StatSoft. Inc., Tulsa, OK, USA, www.statsoft.com). One-way analysis of variance (ANOVA) followed by Tukey’s *post hoc* test was applied to alkaloids content, and differences were considered significant at *p* < 0.05.

## 4. Conclusions

This paper reports valuable information about the phenolic and alkaloid composition of the plant *U. tomentosa.* Using advanced analytical techniques such as UPLC/TQ-ESI-MS and ^13^C-NMR, a total of 32 phenolic compounds have been identified, including hydroxybenzoic acids, hydroxycinnamic acids, flavan-3-ols monomers, procyanidin dimers and trimers, flavalignans, and propelargonidin dimers; these later were reported for the first time in *U. tomentosa*. Concerning alkaloids, eight main previously-reported tetracyclic and pentacyclic alkaloids were quantified in the extract, as well as the detection of other minor ones. For both phenolic and alkaloid extracts, the part of the plant used (leaves, stems, bark and wood) conditions their composition. In addition, as expected, some variability was observed in the content of phenolic compounds among the different origins/locations of the plants.

The extraction procedure used was successful in obtaining added-value extracts from *U. tomentosa*. The phenolic extracts obtained, particularly rich in proanthocyanidins, may exert the health benefits attributed to these compounds, mainly in cardiovascular diseases [24,25] due to its anti-platelet properties and its effect in lipids metabolism and vascular function [26]. In addition, their content in flavalignans (cinchonains) might be extra value as recent reports on insulinotropic effects of cinchonain 1b suggest their possible use in the treatment of diabetes type 2 [27]. Leaves are particularly the part of *U. tomentosa* plants with the highest feasibility for use in the elaboration of standardizing phenolic extracts in a sustainable way, thus susceptible to application in the food, cosmetic and/or pharmaceutical industries.

## Supplementary Materials

Supplementary material including ^1^H- and ^13^C-NMR spectra of alkaloids can be accessed at http://www.mdpi.com/1420-3049/20/12/19875/s1.
