# Supplementary material for: Phenolic Assesment of Uncaria tomentosa L. (Cat’s Claw): Leaves, Stem, Bark and Wood Extracts
Source: Molecules. 2015 Dec 18;20(12):22703–17. doi: 10.3390/molecules201219875 (PMC6332257; doi:10.3390/molecules201219875)
Supplement: Supplementary file 1 [file molecules-20-19875-s001.pdf]

Supplementary Materials: Phenolic Assesment of *Uncaria tomentosa* L. (Cat's Claw): Leaves, Stem, Bark and Wood Extracts

Mirtha Navarro Hoyos, Fernando Sánchez-Patán, Renato Murillo Masis, Pedro J. Martín-Álvarez, William Zamora Ramirez, Maria J. Monagas and Begoña Bartolomé

Table S1. MS/MS parameters for the identified phenolic compounds.

	**MRM Transition (*m/z*)**
Benzoic acid	121 > 77
Salicylic acid	137 > 93
4-Hydroxybenzoic acid	137 > 93
Protocatechuic acid	153 > 109
Gallic acid	169 > 125
Vanillic acid	167 > 152
Syringic acid	197 > 182
p-Coumaric acid	163 > 119
Caffeic acid	179 > 135
Ferulic acid	193 > 134
Isoferulic acid	193 > 134
(+)-Catechin	289 > 245
(−)-Epicatechin	289 > 245
Procyanidin dimers	577 > 289
Procyanidin trimers	865 > 577
Propelargonidin dimers	561 > 289
Flavalignans-cinchonains	451 > 341

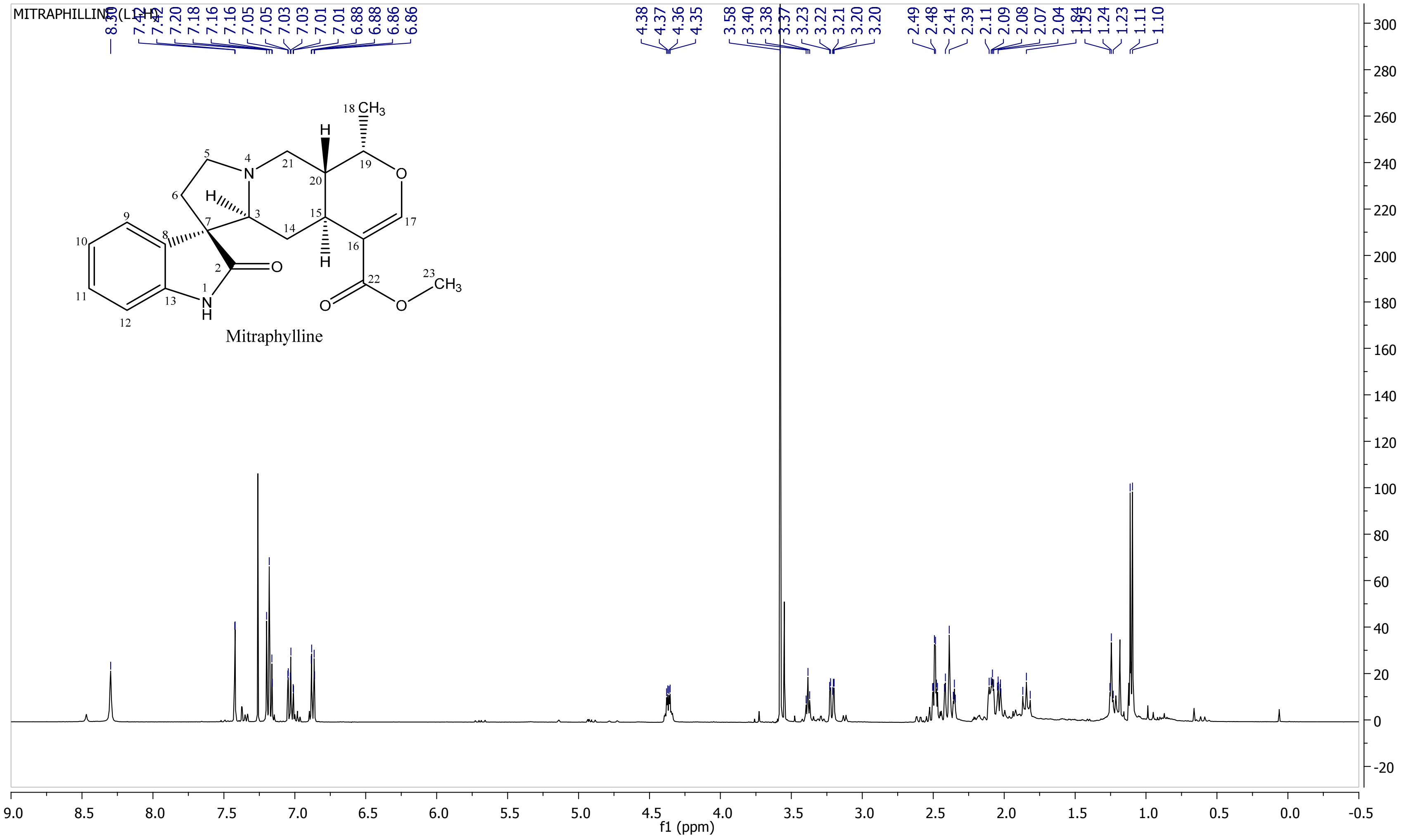

Figure S1. ^{1}H-NMR spectrum (400 MHz, $CDCl_3$) of Mitraphylline (L1-H).

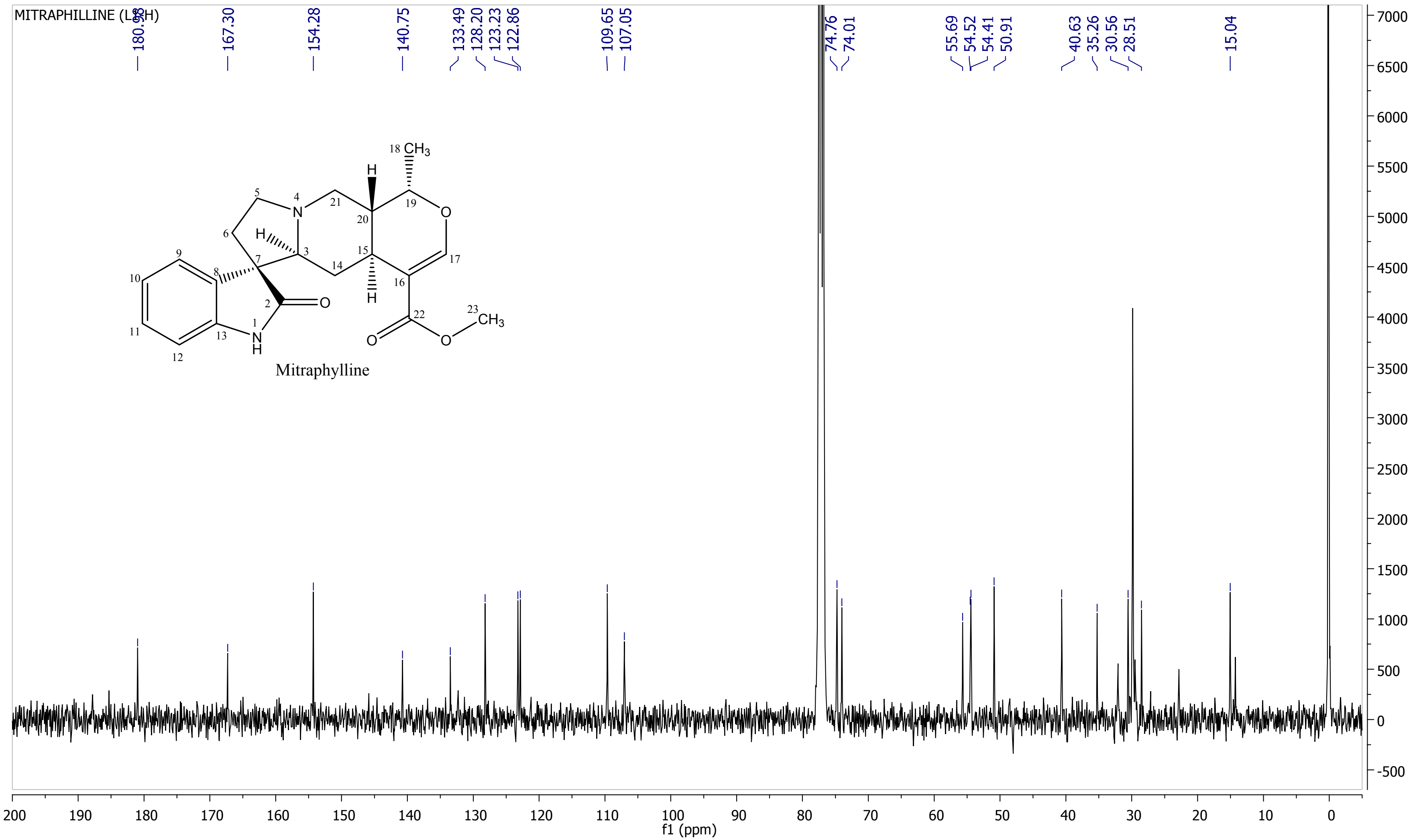

Figure S2. ^{13}C-NMR spectrum (400 MHz, $CDCl_3$) of Mitraphylline (L1-H).

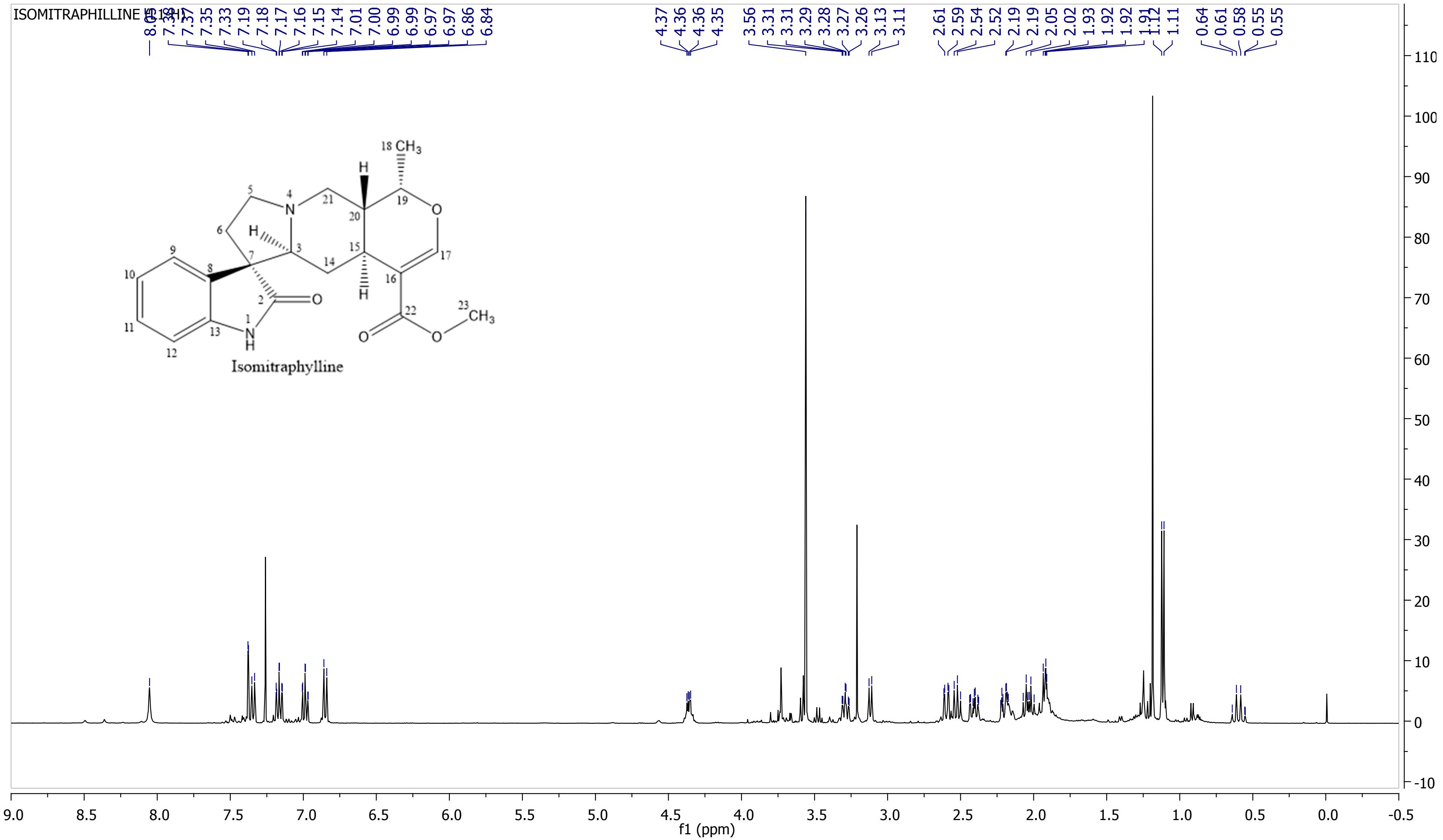

Figure S3. ^{1}H-NMR spectrum (400 MHz, $CDCl_3$) of Isomitraphylline (L1-H).

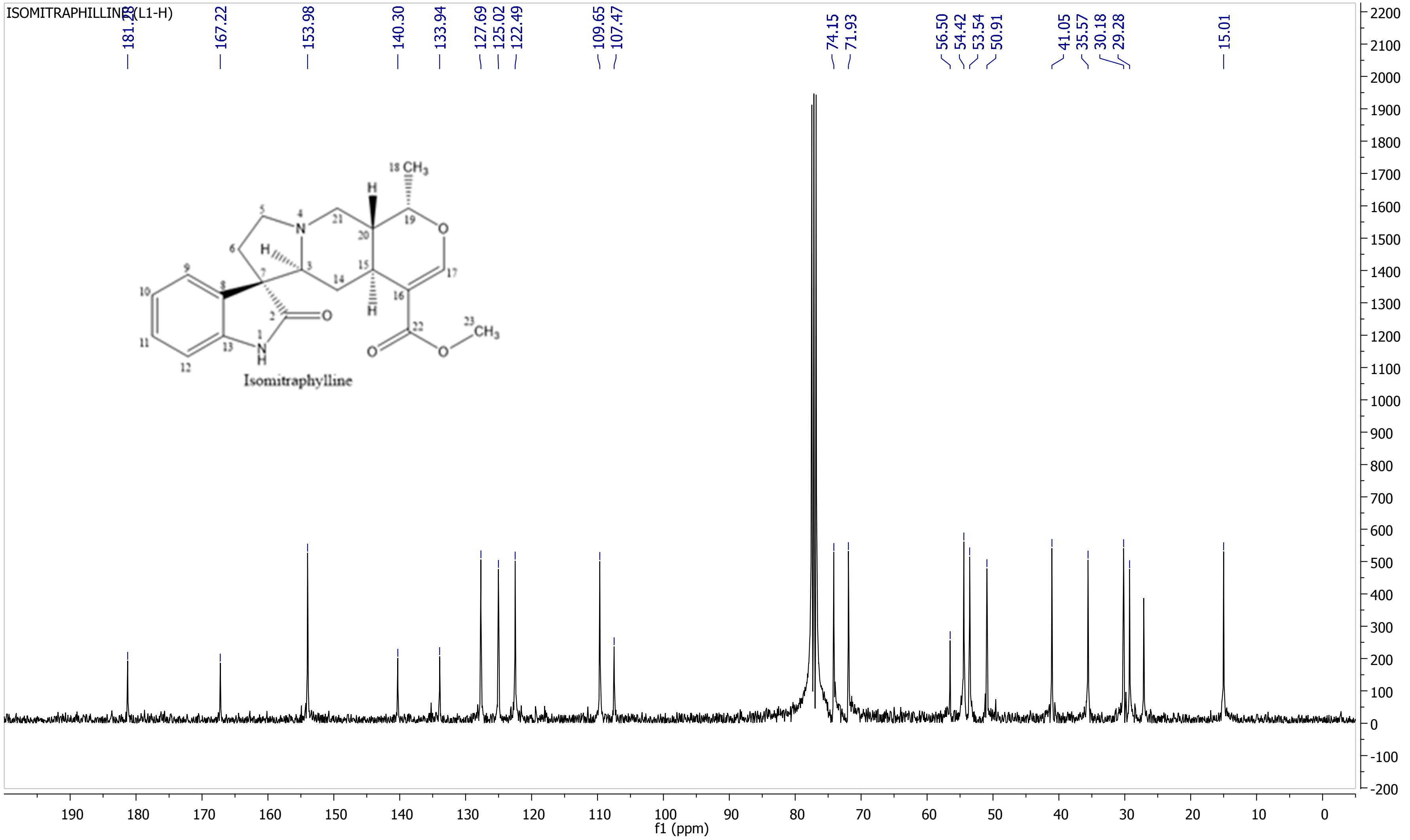

Figure S4. ^{13}C-NMR spectrum (400 MHz, $CDCl_3$) of Isomitraphylline (L1-H).

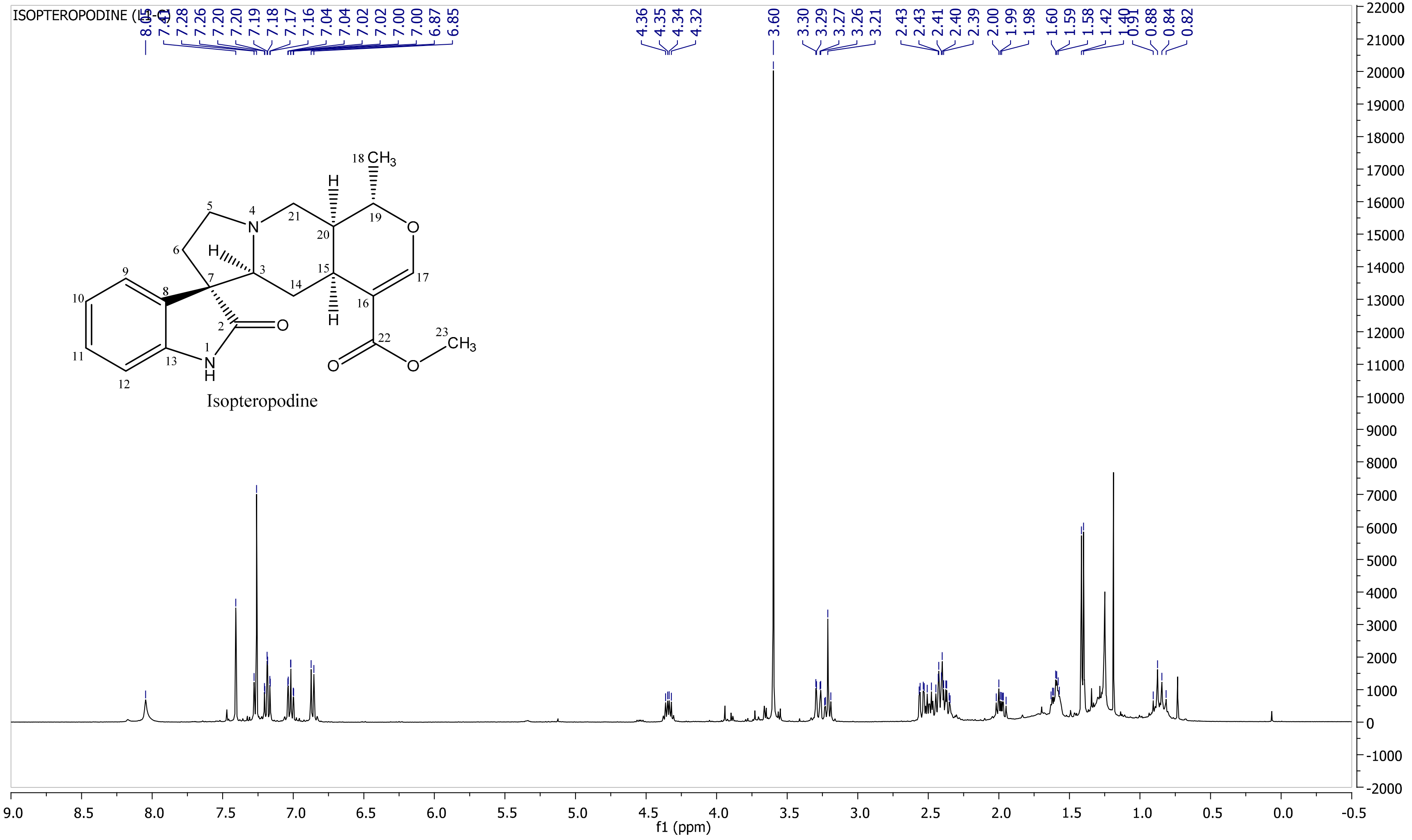

Figure S5. ^{1}H-NMR spectrum (400 MHz, $CDCl_3$) of Isopteropodine (L1-C).

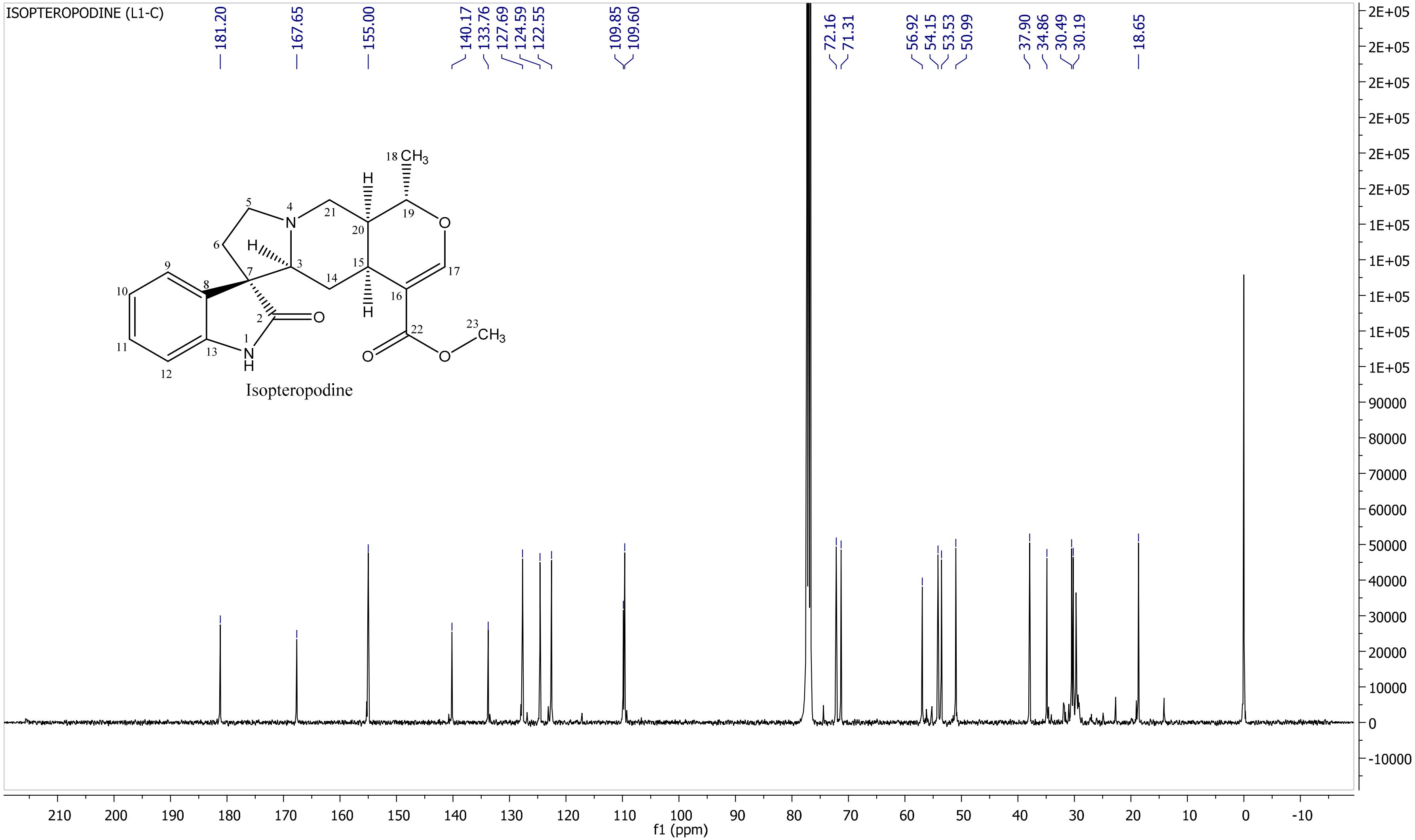

Figure S6. ^{13}C-NMR spectrum (400 MHz, $CDCl_3$) of Isopteropodine (L1-C).

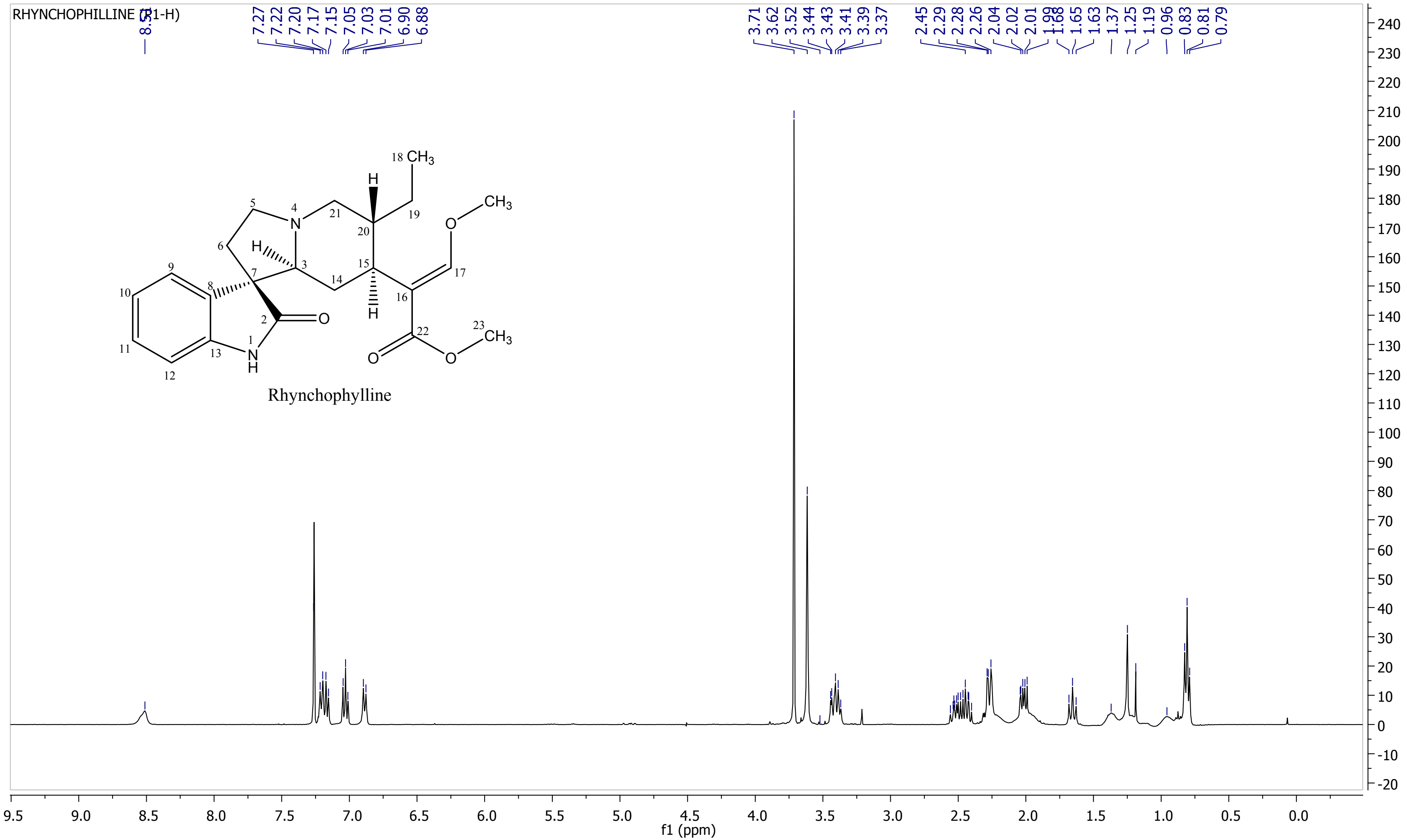

Figure S7. ^{1}H-NMR spectrum (400 MHz, $CDCl_3$) of Rynchophilline (P1-H).

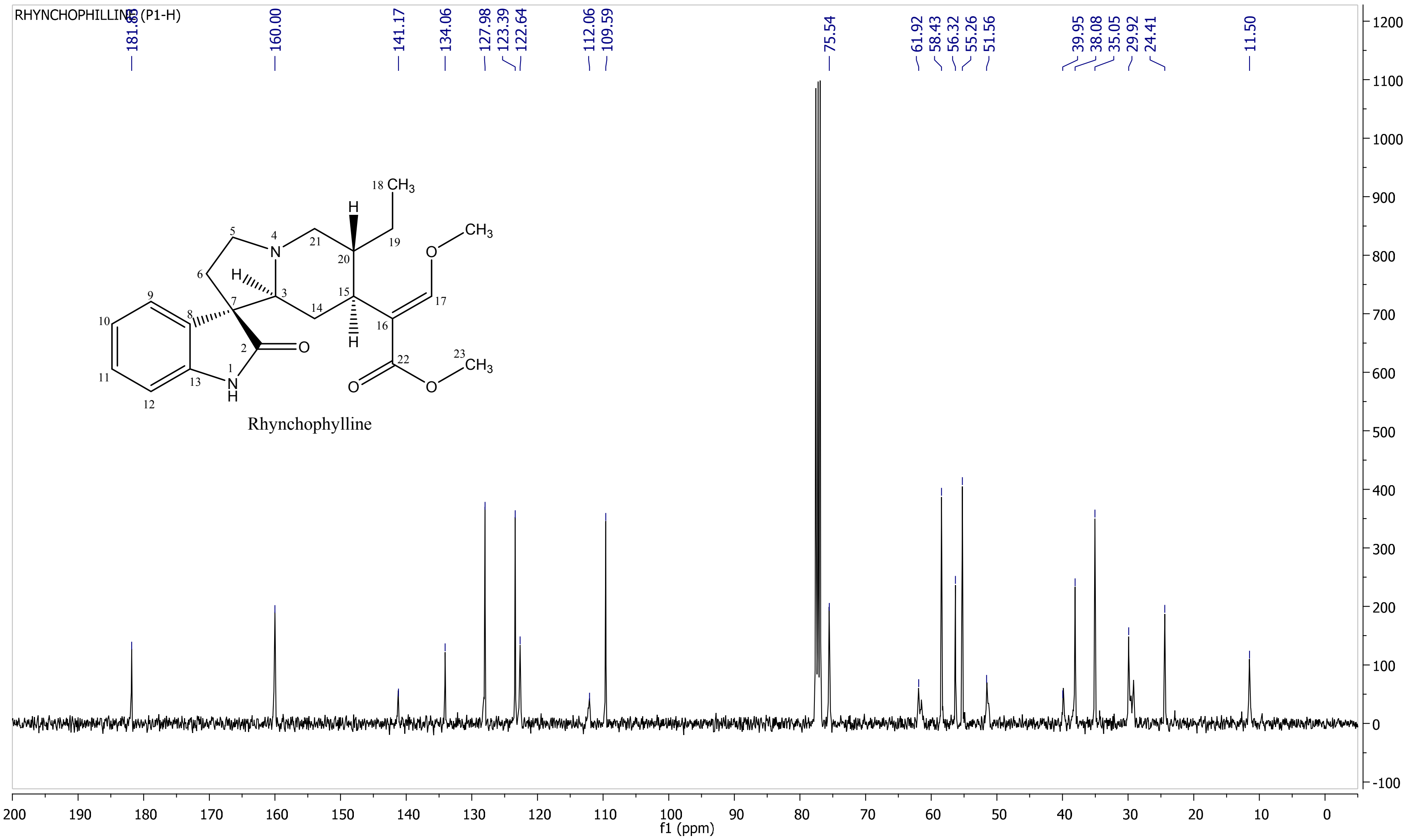

Figure S8. ^{13}C-NMR spectrum (400 MHz, $CDCl_3$) of Rynchophilline (P1-H).

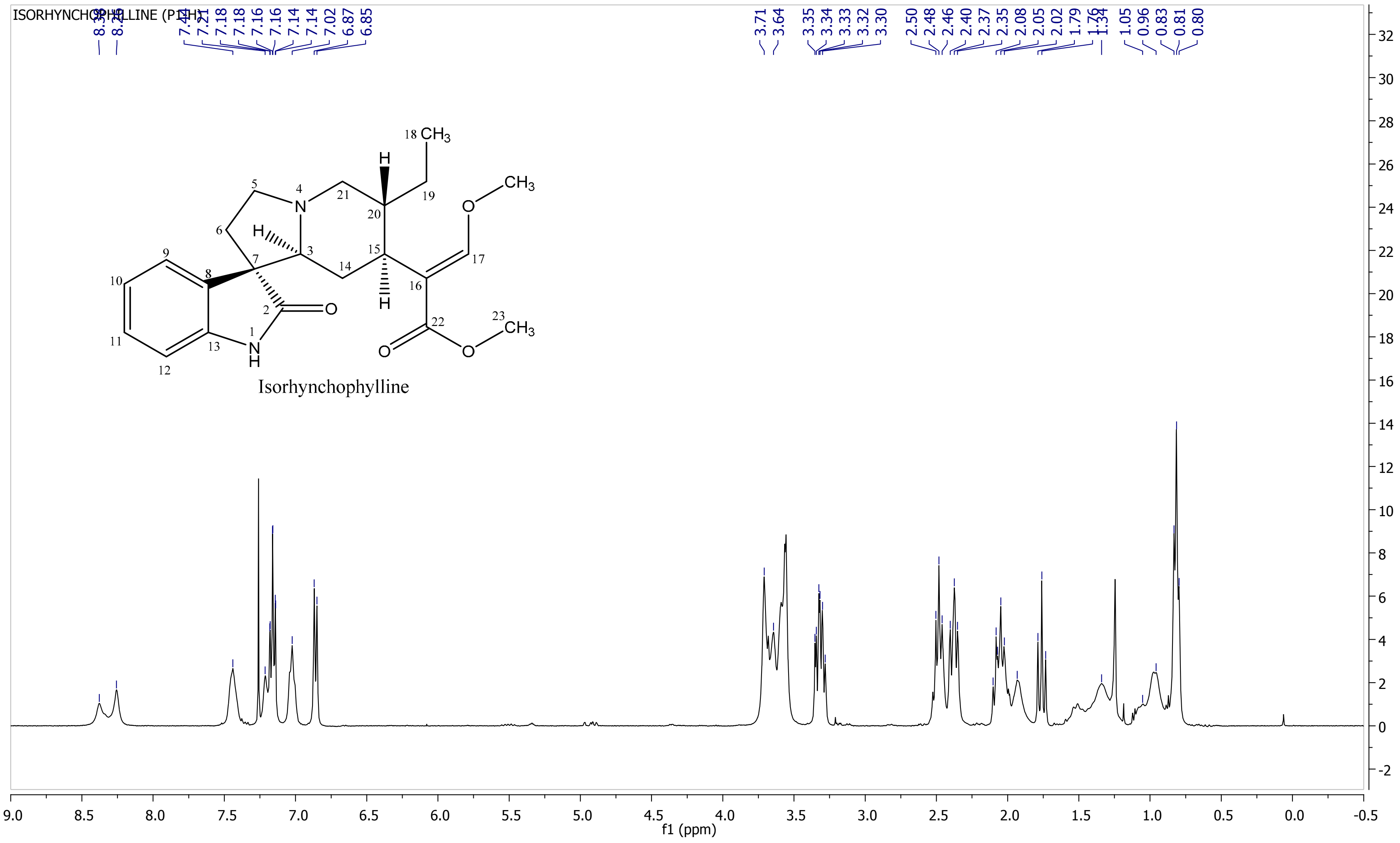

Figure S9. ^{1}H-NMR spectrum (400 MHz, $CDCl_3$) of Isorhynchophylline (P1-H).

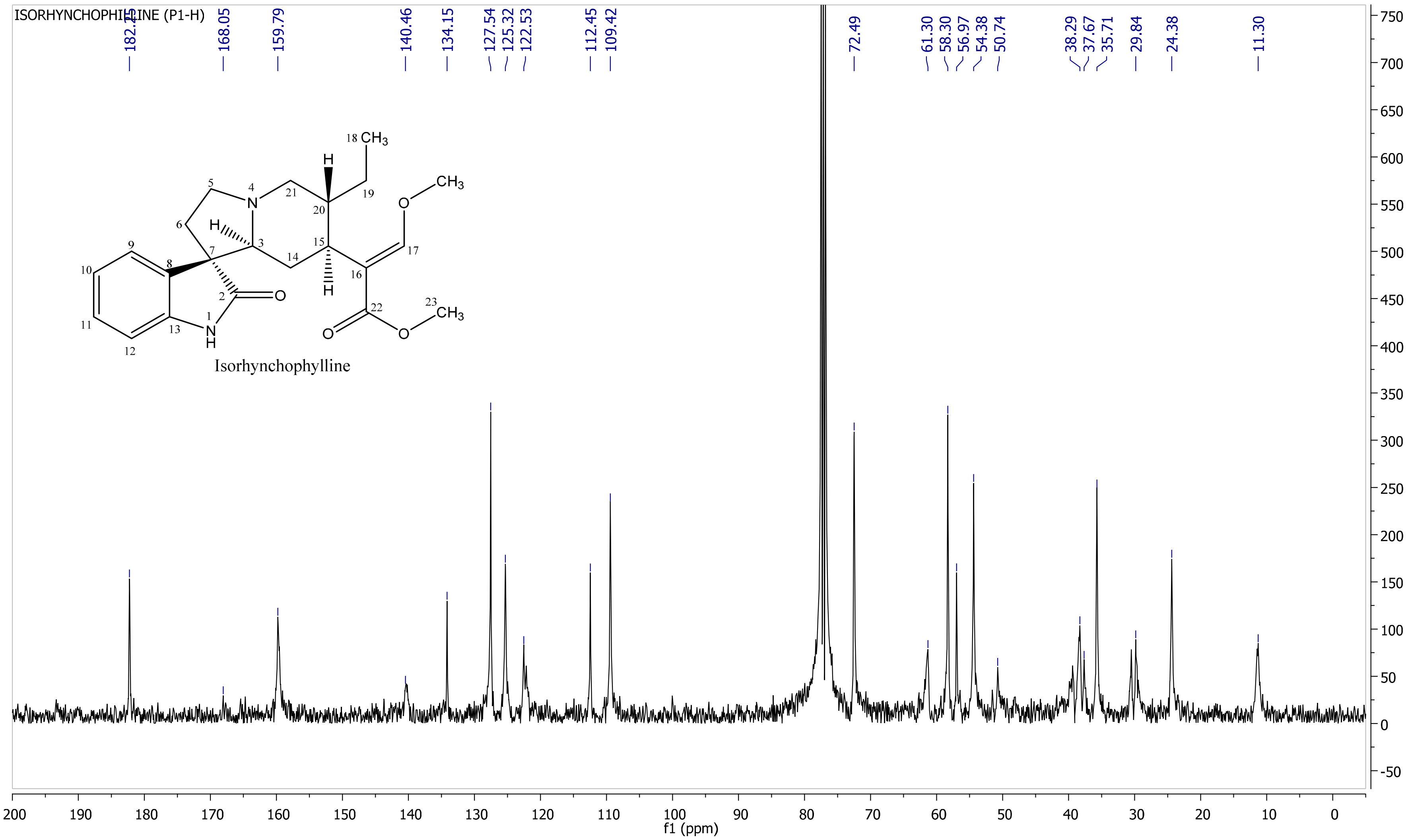

Figure S10. ^{13}C-NMR spectrum (400 MHz, $CDCl_3$) of Isorynchophylline (P1-H).

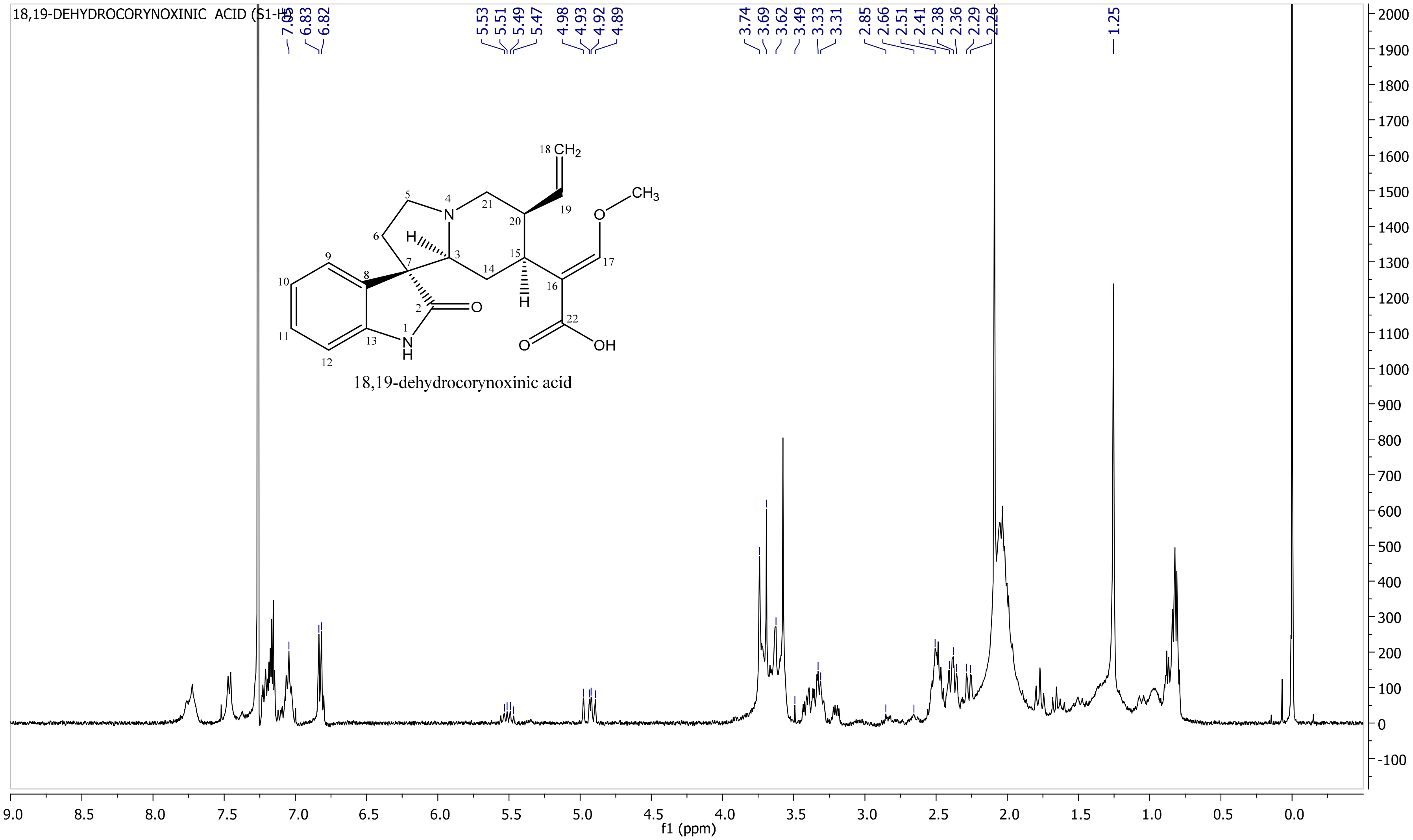

Figure S11. ^{1}H-NMR spectrum (400 MHz, $CDCl_3$) of 18, 19-dehydrocorynoxinoic acid (S1-H).

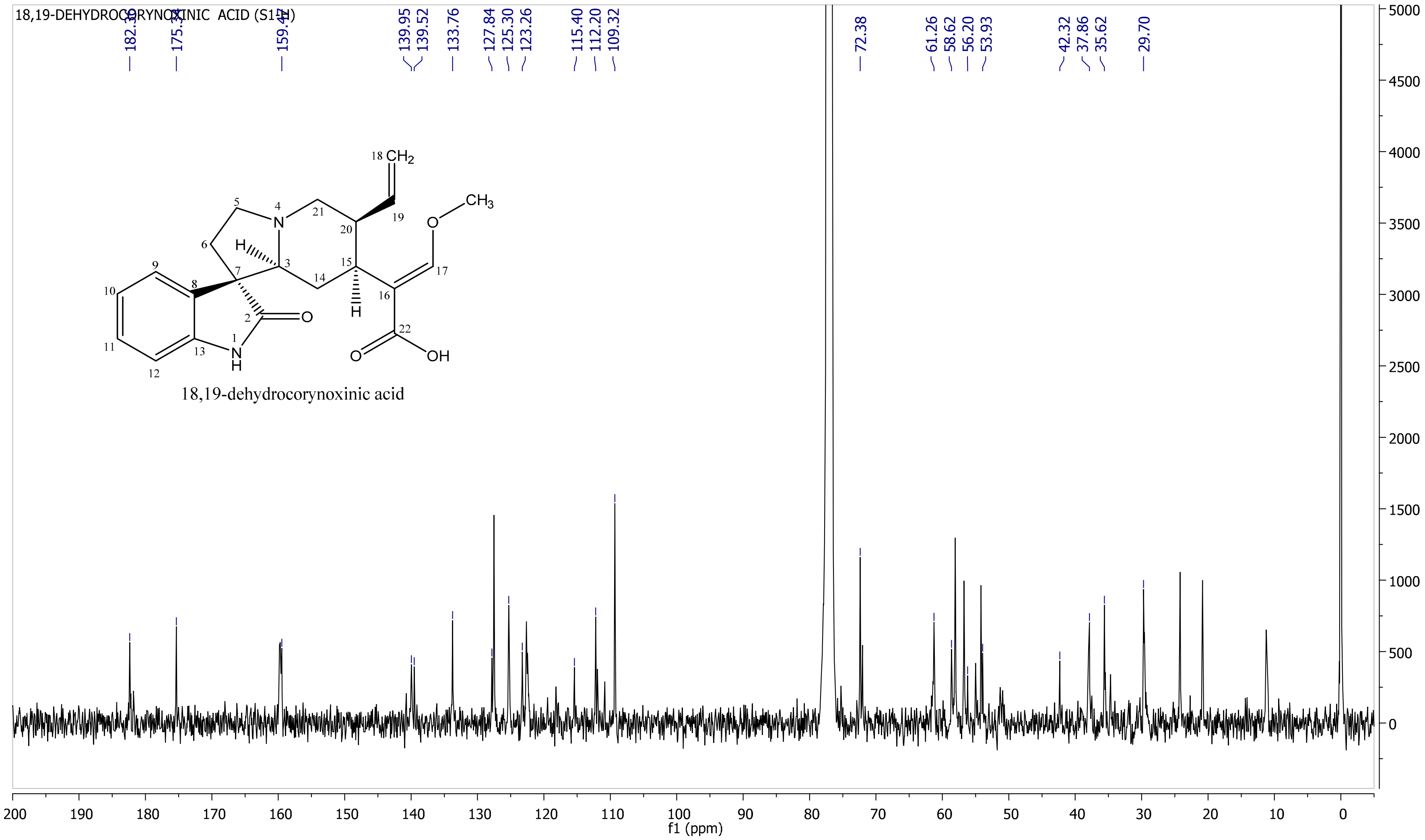

Figure S12. ^{13}C-NMR spectrum (400 MHz, $CDCl_3$) of 18, 19-dehydrocorynoxinoic acid (S1-H).
